# Syphilis Treatment: Systematic Review and Meta-Analysis Investigating Nonpenicillin Therapeutic Strategies

**DOI:** 10.1093/ofid/ofae142

**Published:** 2024-03-13

**Authors:** Gustavo Yano Callado, Maria Celidonio Gutfreund, Isabele Pardo, Mariana Kim Hsieh, Vivian Lin, Mindy Marie Sampson, Guillermo Rodriguez Nava, Tássia Aporta Marins, Rodrigo Octávio Deliberato, Marinês Dalla Valle Martino, Marisa Holubar, Jorge L Salinas, Alexandre R Marra

**Affiliations:** Faculdade Israelita de Ciências da Saúde Albert Einstein, Hospital Israelita Albert Einstein, São Paulo, São Paulo, Brazil; Faculdade Israelita de Ciências da Saúde Albert Einstein, Hospital Israelita Albert Einstein, São Paulo, São Paulo, Brazil; Faculdade Israelita de Ciências da Saúde Albert Einstein, Hospital Israelita Albert Einstein, São Paulo, São Paulo, Brazil; Faculdade Israelita de Ciências da Saúde Albert Einstein, Hospital Israelita Albert Einstein, São Paulo, São Paulo, Brazil; Faculdade Israelita de Ciências da Saúde Albert Einstein, Hospital Israelita Albert Einstein, São Paulo, São Paulo, Brazil; Division of Infectious Diseases & Geographic Medicine, Stanford University, Stanford, California, USA; Division of Infectious Diseases & Geographic Medicine, Stanford University, Stanford, California, USA; Faculdade de Medicina, Centro Universitário de Adamantina, Adamantina, São Paulo, Brazil; Department of Biomedical Informatics, University of Cincinnati College of Medicine, Cincinnati, Ohio, USA; Biomedical Informatics Division, Cincinnati Children's Hospital Medical Center, Cincinnati, Ohio, USA; Faculdade Israelita de Ciências da Saúde Albert Einstein, Hospital Israelita Albert Einstein, São Paulo, São Paulo, Brazil; Division of Infectious Diseases & Geographic Medicine, Stanford University, Stanford, California, USA; Division of Infectious Diseases & Geographic Medicine, Stanford University, Stanford, California, USA; Faculdade Israelita de Ciências da Saúde Albert Einstein, Hospital Israelita Albert Einstein, São Paulo, São Paulo, Brazil; Department of Internal Medicine, University of Iowa Carver College of Medicine, Iowa City, Iowa, USA

**Keywords:** efficacy comparison, nonneurological syphilis, penicillin, syphilis treatment, treatment alternatives

## Abstract

**Background:**

Penicillin's long-standing role as the reference standard in syphilis treatment has led to global reliance. However, this dependence presents challenges, prompting the need for alternative strategies. We performed a systematic literature review and meta-analysis to evaluate the efficacy of these alternative treatments against nonneurological syphilis.

**Methods:**

We searched MEDLINE, the Cumulative Index to Nursing and Allied Health Literature, Embase, Cochrane, Scopus, and Web of Science from database inception to 28 August 2023, and we included studies that compared penicillin or amoxicillin monotherapy to other treatments for the management of nonneurological syphilis. Our primary outcome was serological cure rates. Random-effect models were used to obtain pooled mean differences, and heterogeneity was assessed using the *I*^2^ test.

**Results:**

Of 6478 screened studies, 27 met the inclusion criteria, summing 6710 patients. The studies were considerably homogeneous, and stratified analyses considering each alternative treatment separately revealed that penicillin monotherapy did not outperform ceftriaxone (pooled odds ratio, 1.66 [95% confidence interval, .97–2.84]; *I*^2^ = 0%), azithromycin (0.92; [.73–1.18]; *I*^2^ = 0%), or doxycycline (0.82 [.61–1.10]; *I*^2^ = 1%) monotherapies with respect to serological conversion.

**Conclusions:**

Alternative treatment strategies have serological cure rates equivalent to penicillin, potentially reducing global dependence on this antibiotic.

## Teaser

This review and meta-analysis compared nonneurological syphilis treatments. Results indicate alternative therapies like doxycycline, ceftriaxone, and azithromycin have similar effectiveness and safety as penicillin. Combination therapies demonstrated promise. These findings offer alternatives when penicillin is unavailable, reducing reliance on it.

## Graphical Abstract

**Graphical Abstract:**
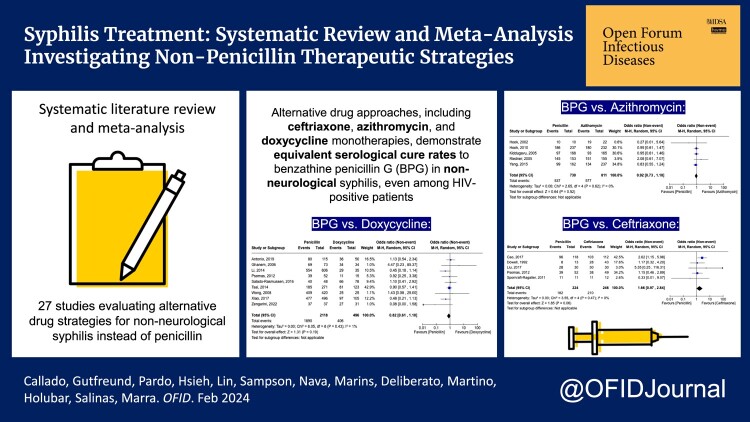

Penicillin is universally recognized as the reference standard therapy for treating syphilis in all stages [1]. Its targeted action on bacterial cell wall synthesis has rendered *Treponema pallidum* highly susceptible, and remarkably there are no documented cases of penicillin resistance in the medical literature [2].

Despite penicillin's widespread use worldwide, its therapeutic merits and established efficacy over many years have made it synonymous with syphilis treatment in practical clinical settings. Many countries have heavily relied on this single-drug approach for managing patients with syphilis. However, this comes with significant challenges. Penicillin shortages in certain centers has led to adverse impacts on syphilis control efforts [3, 4]. Moreover, penicillin allergies also pose challenges to the management of syphilis [5].

The pursuit of penicillin alternatives for syphilis treatment is essential. It ensures that fluctuations in the availability of a single drug do not have a detrimental impact on managing a disease responsible for significant morbidity. In this systematic literature review and meta-analysis, we investigate the efficacy of alternative drug strategies for nonneurological syphilis treatment. By analyzing comparative studies, we explore these alternatives’ effectiveness in managing nonneurological syphilis.

## METHODS

### Systematic Review and Search Strategies

This systematic literature review adhered to both the Preferred Reporting Items for Systematic Reviews and Meta-Analysis (PRISMA) statement [6] and the Meta-analysis of Observational Studies in Epidemiology (MOOSE) guidelines [7]. It was registered on Prospero on 8 September 2023 (registration no. CRD42023458547).

### Search Strategy

Our search strategy was developed with the guidance of a health sciences librarian. We conducted comprehensive searches across multiple databases, including MEDLINE (PubMed), the Cumulative Index to Nursing and Allied Health Literature, Cochrane CENTRAL, Web of Science, Scopus, and Embase. Our search encompassed publications from the inception of each database up to 28 August 2023 (Supplementary Table 1).

This study uses the PICO framework [8]. Focusing on patients diagnosed with nonneurological syphilis (P), the study compares treatment strategies not solely based on penicillin (I) against conventional penicillin or amoxicillin monotherapies (C). Our primary outcome of interest (O) was serological cure rates.

We excluded comments or reviews, noncomparative studies, pilot studies, studies performed solely in children, and those including strictly neurosyphilis, otosyphilis, and/or ocular syphilis. However, studies that presented a reported small proportion of patients (<1%) identified as neurosyphilis were not excluded.

**Figure 1.**
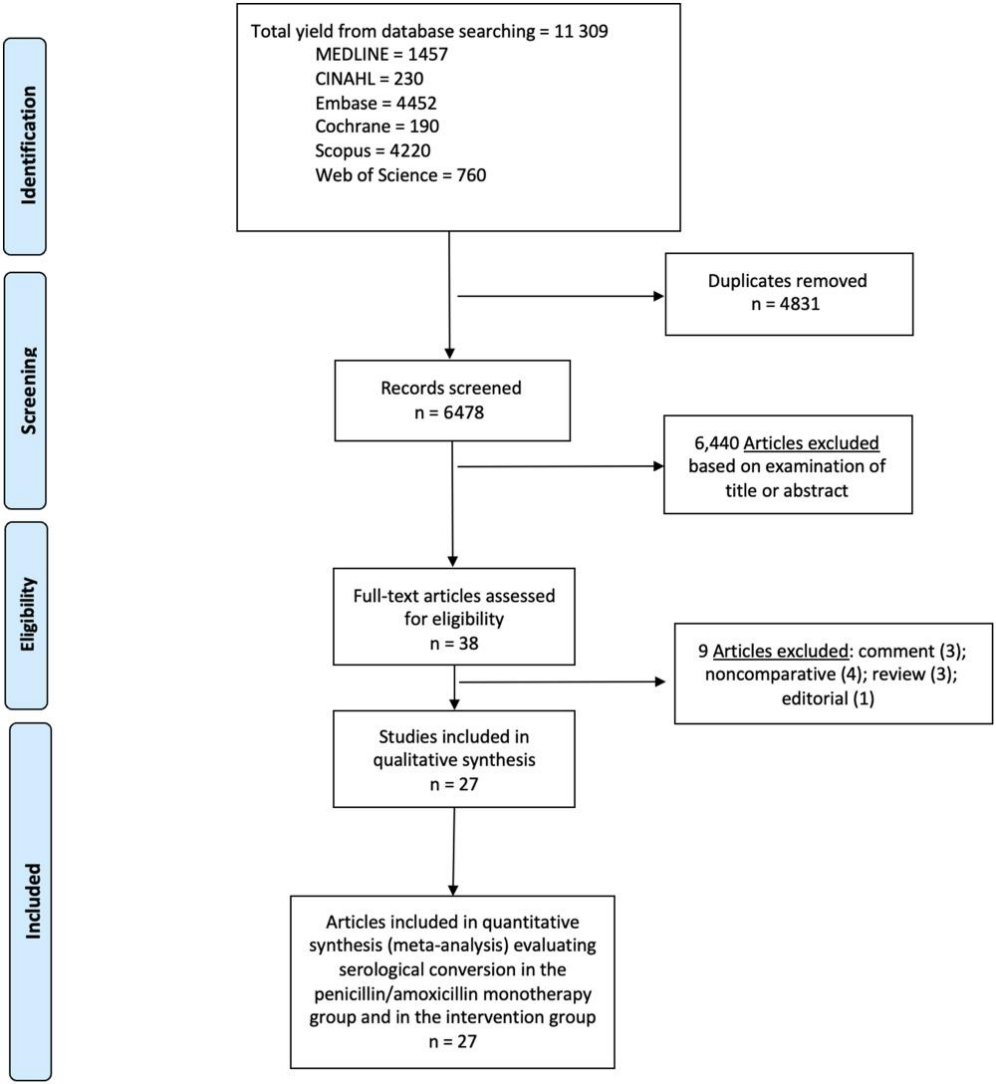
Literature search for articles that evaluated the syphilis treatment alternatives against nonneurological syphilis. Abbreviation: CINAHL, Cumulative Index to Nursing and Allied Health Literature.

All titles and/or abstracts were examined (G. Y. C. and A. R. M.), and those deemed unsuitable were excluded. Disparities were resolved through discussion. After this first evaluation, all the remaining articles were fully read, and the studies that met the inclusion criteria were included in the systematic review (Figure 1).

### Data Abstraction and Quality Assessment

Of 10 independent reviewers (G. Y. C., M. C. G., I. P., M. K. H., V. L., M. M. S., G. R. N., T. A. M., R. O. D., and A. R. M.), 2 abstracted data from each article using a standardized abstraction form (Supplementary Form 1). We recorded the publication year, study period, design, population, setting, analyzed drugs, dosage, duration of the compared strategies, serological response definition, cure rates, and adverse effects associated with treatment.

We used the Downs and Black scale [9] to assess the quality of the studies included in our review. Each article underwent a thorough evaluation, with all the original scale's questions being addressed, and we calculated a total score. We made a modification to question 27, substituting the multiple-choice options with a simple yes/no response format. The maximum achievable score on this scale was 28. Our reviewers independently assessed the individual components’ quality, and any disparities were resolved through discussion.

### Patient Consent Statement

The present investigation is a systematic literature review and meta-analysis of published data, so no patient-informed consent was required.

### Statistical Analysis

Our outcome of interest was a serological response according to the definition presented by the analyzed article. For all the studies, we considered information referring to the longest reported follow-up. We evaluated responses to the compared therapy strategies by using a random-effects model. This model estimated pooled odds ratios (ORs) and their corresponding 95% confidence intervals (CIs). We determined weights for the analysis in accordance with the approach outlined by DerSimonian and Laird [10]. We performed stratified analyses considering each studied drug, study design, publication period, location, nontreponemal/treponemal tests applied, human immunodeficiency virus (HIV) serological status, and other variables (Supplementary Table 2).

Heterogeneity between studies was evaluated using the *I*^2^ statistic and the Cochran Q statistic test. We used Cochrane Review Manager (RevMan), Web edition 4.12.0. Publication bias was evaluated by visual inspection of funnel plots with RevMan (Supplementary Figure 2) and was also evaluated by applying the Egger test with Comprehensive Meta-Analysis software, version 4 (Biostat).

## RESULTS

Of 38 articles reviewed in further detail, 27 met the inclusion criteria for this systematic literature review [11–37] (Figure 1). Ten were randomized clinical trials [11, 13, 16, 18, 19, 22, 23, 25, 26, 29], 16 were retrospective cohort studies [12, 14, 15, 17, 20, 21, 24, 27, 28, 30–34, 36, 37], and 1 was a prospective cohort study [35]. Twelve were conducted in East Asia [11, 13, 14, 21–23, 28, 31, 33–36], 7 in North America [15, 17–19, 26, 29, 32], 5 in Europe [16, 24, 27, 30, 37], 2 in Africa [20, 25] and 1 in South America [12]. Five compared benzathine penicillin G (BPG) or penicillin derivative (amoxicillin) monotherapy with a combined therapy [11, 14, 16, 26, 36], and 21 compared penicillin with a different drug monotherapy [12, 13, 15, 17–19, 21–25, 27–35, 37]. As for the control group, only 1 study did not use penicillin, using amoxicillin instead [11]. Kiddugavu et al [20] had the only study to include 3 arms (penicillin monotherapy, azithromycin monotherapy, and combined penicillin plus azithromycin). Among the 22 studies that included a monotherapy arm, 8 studied doxycycline [12, 17, 21, 27, 31, 32, 34, 37], 5 studied ceftriaxone [13, 15, 22, 29, 30], 5 studied azithromycin [18–20, 25, 35], 2 studied minocycline [28, 33], 1 studied cefixime [23], and 1 studied both doxycycline and ceftriaxone separately [24].

The included studies used a variety of serological tests. Regarding the treponemal tests, 6 used *T pallidum* particle agglutination assay [13, 14, 23, 25, 33, 34], 5 used *T pallidum* hemagglutination assay [11, 20, 26, 31, 37], 3 used fluorescent treponemal antibody absorption test [17–19], 2 used microhemagglutination assay for *T pallidum* [15, 29], 7 used >1 test [16, 21, 24, 27, 30, 32, 35], and 4 did not report this information [12, 22, 28, 36]. Of the nontreponemal tests, 15 studies used rapid plasma reagin (RPR) [11, 13, 14, 17–19, 21, 25, 27, 31–35, 37], 5 used the VDRL test [12, 16, 24, 26, 30], 3 used toluidine red unheated serum test (TRUST) [20, 22, 23], other 3 used either RPR or VDRL [15, 28, 29], and 1 used either RPR or TRUST [36].

Among the 27 studies, 10 included only HIV-negative patients [13, 19, 21–23, 28, 32–34, 36], 7 included only HIV-positive patients [11, 12, 15, 27, 30, 31, 35], and 10 included patients regardless of HIV serological status [14, 16–18, 20, 24–26, 29, 37]. Twenty-six of the 27 studies reported a specific definition of serological cure [11–21, 23–37]; 24 studies defined cure as ≥4-fold (or 2 dilutions) decline in titers of a nontreponemal test [11–21, 23, 25–27, 29–37]. However, these studies varied in the specified time to cure. Six studies specified 6 months [13, 15, 16, 19, 21, 32]; 4 studies, 12 months [11, 14, 17, 35]; 3 studies, any time between 6 and 12 months [12, 31, 34]; and 1 study each, 9 months [25], 10 months [20], any time between 12 and 24 months [29], and 24 months [32]. Seven studies did not specify the time to cure [18, 23, 26, 27, 30, 33, 36]. One study defined cure as a nonreactive nontreponemal test result after 24 months [28], and another considered cure as a decrease in VDRL ≤1:4 [24]. One study did not provide a definition of serological cure [22].

Most studies (21 of 27) found no difference between penicillin monotherapy and other strategies in regard to serological cure [11, 12, 15, 17–19, 21–27, 29–35, 37], 5 studies found a difference [13, 14, 16, 28, 36], and 1 study reported a difference in cases with a high initial titer but no difference in patients with low initial titer [20]. All 5 studies that reported differences favored an alternative regimen other than penicillin monotherapy: ceftriaxone [13], penicillin plus doxycycline [14], penicillin plus ceftriaxone plus doxycycline [16], minocycline [28], or penicillin plus ceftriaxone [36].

Regarding adverse effects related to medications, 18 studies reported no data [12, 14, 15, 17, 20–22, 24, 26–34, 37]. The effects reported by the other 9 studies were gastrointestinal (6 studies) [11, 18, 23, 25, 35, 36], followed by the Jarisch-Herxheimer reaction (4 studies) [11, 13, 18, 23], and rash (2 studies) [11, 36]. A summary of the reported adverse effects can be seen in Table 1. No studies reported serious adverse effects; overall, there was no significant difference in medication-related adverse effects between study arms.

**Table 1.** Summary of Study Characteristics

Authors (Year; Location) [Follow-up Period]	Study Design	Treponemal/Nontreponemal Tests	Control [Penicillin or Amoxicillin] and Intervention Groups [Other Drugs] (No. of Participants)	Age Median y; % Male; % HIV Positive)	Stage of Syphilis (No. Control, No. Intervention)	Regimens	Adopted Definition of Cure/Serological Response	Difference Among Treatments and Serological Response	Analyzed Adverse Drug Events	Reinfection	Downs and Black Score
Ando et al [11] (2023; Tokyo, Japan) [Aug 2018–Feb 2022]	RCT	TPHA/RPR	Amoxicillin (56)	39 (IQR, 33–46); 100; 100	Primary (2, 3); secondary (30, 27); early latent (17, 18); late latent (7, 8)	Amoxicillin 500 mg, thrice daily by mouth for 14 d for early and 28 d for late syphilis	≥4-Fold decline or negative conversion in RPR titer within 12 mo of treatment	ITT analysis (overall syphilis): 3 mo: 32 (57.1%) and 41 (73.2%) [*P* < .75]; 6 mo: 42 (75.0%) and 47 (83.9%) [*P* < .44]; 12 mo: 48 (85.7%) and 51 (91.1%) [*P* = .23]	Overall adverse effects: 10/56; J-H reactions: 7/56; drug rashes: 3/56; nausea: 2/56; diarrhea: 0	Defined and presented cases: 4 participants (3.6%) exhibited subsequent ≥4-fold increase during follow-up; all 4 presented with systemic rashes consistent with secondary syphilis and engaged in high-risk sexual behavior, indicating that they were cases of reinfection	25
			Amoxicillin + probenicid (56)	39 (IQR, 30.5–46.5); 100; 100		Amoxicillin 1000 mg + probenecid 250 mg, thrice daily, by mouth for 14 d for early and 28 d for late syphilis			Overall adverse effects: 14/56; J-H reactions: 8/56; drug rashes: 4/56; nausea: 1/56; diarrhea: 3/56		
Antonio et al [12] (2019; São Paulo, Brazil) [Sep 2014–Dec 2016]	Retrospective cohort study	NR/VDRL	BPG (115)	44 (IQR, 37–50); 99; 100	Primary (3, 1); secondary (4, 3); early latent (65, 26); late latent (8, 3); and unknown latent (35, 17)	BPG ≥1 2.4 MIU intramuscular dose for early stages and 3 weekly 2.4 MIU intramuscular doses for late latent and unknown-duration stages	Nonreagent VDRL or ≥4-fold reduction in VDRL titers measured 6–12 mo after treatment	At 6–12 mo: 80 (69.6%) and 36 (72%) [*P* = .08]	NR	Did not define or present no. of cases; study could not distinguish between true treatment failure and reinfection	20
			Doxycycline (50)	49 (IQR, 43–56); 99; 100		Doxycycline ≥100 mg by mouth, twice daily for 14 d for early stages 28 d for late latent and unknown-duration stages					
Cao et al [13] (2017; Jiangsu, China) [Nov 2013–Nov 2015]	RCT	TPPA/RPR	BPG (118)	60.2% of participants 18–35 y; 50; 0	Primary (25, 20); secondary (63, 72); early latent (30, 20)	BPG 2.4 MIU intramuscularly, once weekly for 2 wk	≥4-Fold decline in RPR titer within 6 mo of treatment	Yes; 3 mo: 86/115 (74.8%) and 86/110 (78.2%); 6 mo: 92 (78.0%) and 101 (90.2%) [*P* < .05]; 12 mo: 96 (81.4%) and 103 (92.0%) [*P* < .05]	J-H reaction: 31.4%	Did not define but presented cases; 2 patients (0.9%) were reinfected (not clear which group)	21
			Ceftriaxone (112)		51.8% of participants 18–35 y; 42.9; 0		Ceftriaxone 1.0 g intravenously, once daily for 10 d			J-H reaction: 41.1%	
Chen [14] (2023; Taipei, Taiwan) [Jan 2018–Mar 2022]	Retrospective cohort study	TPPA/RPR	BPG (347 with 391 episodes)	37 (IQR, 31–45); 100; 100	Primary + secondary (73 episodes, 95 episodes); early latent (318 episodes, 212 episodes)	BPG 2.4 MIU intramuscular, single dose	≥4-Fold decline in RPR titer within 12 mo of treatment	Yes; ITT analysis (overall syphilis): 3 mo: 155 (54.2%) and 148 (64.1%) episodes [*P* < .02]; 6 mo:: 267 (68.3%) and 247 (80.5%) episodes [*P* < .01]; 12 mo: 275 (70.3%) and 244 (79.5%) episodes [*P* = .006]	NR	Defined and presented cases: defined as occurrence of newly developed symptoms and/or 4-fold increases in RPR titers; of 698 syphilis episodes, 18.3% (128/698) were repeatedly included due to reinfection; a higher proportion of syphilis episodes in patients receiving BPG + doxycycline were from repeatedly included patients, but with no significant between-group differences in the median intervals between recurrent episodes	21
			BPG + doxycycline (223 with 307 episodes)	35 (IQR, 31–43); 100; 99.6		BPG 2.4 MIU intramuscular (single dose) + doxycycline 100 mg, twice daily for 7 d					
Dowell et al [15] (1992; Texas, USA) [Nov 1989–Feb 1991]	Retrospective cohort study	MHA-TP/RPR (for serum specimens) and VDRL (for CSF)	BPG (13)	30.2; 84.6; 100	Presumed latent (13, 30); documented latent (0, 6); neurosyphilis (0, 7)	BPG 2.4 MIU intramuscular once weekly for 3 wk	≥4-Fold decline in serum RPR titer sustained during follow-up period	At ≥6 mo: 8 (61.5%) and 28 (65.1%)	NR	Did not define or present no. of case;: relapse cannot definitively be distinguished from reinfection	17
			Ceftriaxone (43)	34.9; 88.4; 100		Ceftriaxone 1.0 g (or rarely 2.0 g) intravenously once daily for 10–14 d or 1.0 g intramuscular on weekdays until 10–14 doses were administered					
Drago et al [16] (2016;( Italy) [Jan 2010–Dec 2013]	RCT	TPHA and a reactive enzyme immunoassay IgM and IgG/VDRL	BPG (38)	31 (IQR, 21–43); 73.7; 2.6	Primary (15, 7); secondary (12, 6); early latent (8, 9); late latent (3, 9)	For primary, secondary, or early latent syphilis, intramuscular BPG 2.4 MIU (1 dose); for late latent syphilis, intramuscular BPG 2.4 MIU weekly for 3 wk	3–4-Fold decline in VDRL titer within 6 mo after therapy	Yes; 3 mo: 0 (0%) and 11/22 (50%); 6 mo: 13 (34.2%) and 20/22 (90.9%); 12 mo: 26 (68.4%) and 22/22 (100%)	None found	NR	20
			BPG + ceftriaxone + doxycycline (31)	36 (IQR, 20–68); 77.4; 16.1		For primary, secondary and early latent syphilis: intramuscular BPG 2.4 MIU (1 dose) + intramuscular ceftriaxone 1.0 g once daily for 10 d + oral doxycycline 100 m, twice daily for 20 d; for late latent syphilis: intramuscular BPG 2.4 MIU weekly for 3 wk + intramuscular ceftriaxone 1.0 g once daily for 10 d + or doxycycline 100 mg twice daily for 20 d			None found		
Ghanem et al [17] (2006; Maryland, USA) [Oct 1993–Jan 2000]	Retrospective cohort study	FTA-Abs/RPR	BPG (73)	34 (IQR, 27–39); 44.1; 13.7	Primary (15, 6); secondary (44, 17); early latent (14, 11)	Intramuscular BPG 2.4 MIU, single dose	≥4-Fold drop in RPR titer 270–400 d after treatment	400 d after treatment: 69 (94.5%) 34 and (100%) [*P* = .20]	NR	Did not define, but presented no. of cases; patients with treatment failure deemed secondary to reinfection were excluded (0.0%)	18
			Doxycycline (34)	34 (IQR, 27–38); 43.8; 5.9		Doxycycline 100 mg by mouth, twice daily for 14 d					
Hook et al [18] (2002 (Alabama; USA) [Oct 1995–Dec 1997]	RCT	FTA-Abs/RPR	BPG (21)	29 (Range, 18–46); 57.1; 9.5	Primary (11, 8, 11); secondary (6, 9, 9); early latent (4, 4, 12)*	Intramuscular BPG 2.4 MIU, once weekly for 1 or 2 wk	Resolution of all signs and symptoms of syphilis, including all suspicious lesions present at baseline, and either a negative RPR titer or a 4-fold decrease in RPR titer	At 3 mo: 12/14 (85.7%), 15/17 (88.2%), and 20/28 (71.4%); 6 mo: 10/12 (83.3%), 16/17 (94.1%), and 20/26 (76.9%); 9 mo: 9/9 (100%), 14/14 (100%), and 19/24 (79.2%;) 12 mo: 10/10 (100%), 14/14 (100%), and 19/22 (86.4%)	J-H reaction: 24%; nausea: 5%	NR	21
			Azithromycin 2 g (21)	33 (Range, 18–56); 61.9; 0		Azithromycin 2.0 g by mouth, single dose			J-H reaction: 17%; vomiting: 2%; nausea: 13%; and diarrhea: 10%		
			Azithromycin 4 g (32)	28 (Range, 18–49); 50.0; 3.1		Azithromycin 2.0 g by mouth, 2 doses administered 6–8 d apart					
Hook et al [19] (2010; Alabama, USA) [Jun 2000–Mar 2007]	RCT	FTA-Abs/RPR	BPG (262)	27; 66.4; 0	Primary (73, 63); secondary (120, 117); early latent (69, 74)	Intramuscular BPG 2.4 MIU	Decrease in RPR titer at 6-mo follow-up visit of ≥2 dilutions (4-fold) compared with initial RPR titer	ITT analysis (overall syphilis): 3 mo: 187/247 (75.7%) and 177/238 (74.4%); 6 mo: 186/237 (78.5%) and 180/232 (77.6%) Per protocol: 3 mo: 173/231 (74.9%) and 160/218 (73.4%); 6 mo: 180/228 (78.9%) and 169/218 (77.5%)	Administration related: 9.8%	Did not define or present no. of cases; patients deemed to be reinfected with syphilis (no. NR) were retreated with penicillin therapy	22
			Azithromycin (255)	27; 54.5; 0		Azithromycin 2.0 g by mouth, single dose			Administration related: 4.9%		
Kiddugavu et al [20] (2005; Rakai, Uganda) [NR 1994–NR 1998]	Retrospective cohort study	TPHA/TRUST	BPG (168)	31.5%; 30–39 y; 35.1; 19.0	Primary/secondary/early latent (total = 133); late latent (total = 818)	Intramuscular BPG 2.4 MIU, single dose	4-Fold reduction in TRUST titers or seroreversion at 10 mo	Yes in cases with a high initial TRUST titer; no in low-titer infections; 10 mo: 97 (57.8%), 93 (56.4%), and 390 (63.0%)	NR	Did not define or present no. of cases; all seropositive study respondents were offered screening and retreatment every 10 mo, which enabled treatment of all reinfections	18
			Azithromycin (165)	29.1%; 30–39 y; 50.3; 18.2		Oral azithromycin 1.0 g, 1 dose					
			BPG + azithromycin (619)	34.4%; 30–39 y; 43.8; 22.0		Oral azithromycin 1.0 g + intramuscular BPG 2.4 MIU (each single dose)					
Li and Zheng [21] (2014; Peking, China) [Dec 2000–Dec 2011]	Retrospective cohort study	FTA-Abs and TPPA/RPR	BPG (606)	29.0%; 27–33 y; 53.3; 0	Primary (80, 6); secondary (320, 14); early latent (206, 15)	Intramuscular BPG 2.4 MIU weekly for 2 wk	≥4-Fold decline by 6 mo after treatment	6 mo: 554 (91.4%) and 29 (82.9%) [*P* = .16]	NR	NR	16
			Doxycycline or tetracycline (35)	31.4%; 27–33 y; 40.0; 0		Doxycycline 100 mg by mouth, twice daily for 14 d, or tetracycline 500 mg by mouth, 4 times daily for 14 d					
Liu et al [22] (2017; Tangshan, China) [May 2014–May 2015]	RCT	NR/TRUST	Procaine penicillin G (30)	34.6 (SD ± 9.4); 46.7%; 0%	Primary (total = 12); secondary (total = 13); early latent (total = 5)	Intramuscular procaine penicillin G 800 000 IU, once daily for 15 d	NR	At 3 mo: 7 (23.3%) 13 and (43.3%) [*P* = .1]; 6 mo: 18 (60%) and 23 (76.7%) [*P* = .17]; 12 mo: 28 (93.3%) and 30 (100%) [*P* = .15]	NR	NR	16
			Ceftriaxone (30)	35.4 (SD ± 9.5); 53.3%; 0%		Intravenous ceftriaxone 1.0 g, once daily for 10 d					
Pei et al [23] (2021; Shandong, China) [May 2018–Apr 2020]	RCT	TPPA/TRUST	BPG (30)	38.1 (SD ± 14.5); 43.3%; 0%	Primary (13, 18); secondary (17, 12)	Intramuscular BPG 2.4 MIU, once weekly for 2 wk	Rash subsides and TRUST titer turns negative or drops ≥4-fold	At 3 mo: 0 and 0; 6 mo: 1 (3.3%) and 2 (6.7%); 12 mo: 12 (40%) and 16 (53.3%)	J-H reaction: diarrhea	NR	22
			Cefixime (30)	37.8 (SD ± 14.0); 53.3%; 0%		Cefixime 100 mg by mouth, twice daily for 15 d			Gastrointestinal reactions (nausea/emesis)		
Psomas et al [24] (2012; Montpellier, France) [Oct 1993–Dec 2007]	Retrospective cohort study	FTA-Abs and TPHA/VDRL	BPG (52)	42 (IQR: 23–49); 98.3%; 80.2%	Primary (6, 1, 2); secondary (34, 47, 8); latent syphilis (12, 1, 5); neurosyphilis (1, 18, 1)*	Intramuscular BPG 2.4 MIU at 1-wk intervals up to 3 weeks, depending on the syphilis stage	Decrease in serum antibody rate; VDRL result ≤1:4.	39 (75.0%,) 38 (77.6%), and 11 (73.3%)	NR	NR	16
			Ceftriaxone (49)			Intravenous ceftriaxone 1 or 2 g daily for 14–21 d					
			Doxycycline (15)			Doxycycline 100 mg by mouth, 2 or 3 times daily for 14–21 d					
Riedner et al [25] (2005; Mbeya, Tanzania) [Sep 2000–Sep 2003]	RCT	PCR/RPR	BPG (165)	53.3% of participants ≥25 y; 24.8; 52.7	Primary (14, 11); latent (151, 152)	Intramuscular BPG 2.4 MIU, single dose	Decrease in RPR titer by ≥2 dilutions before or at 9-mo follow-up examination	At 3 mo: 91/153 (59.5%) and 92/155 (59.4%); 6 mo: 125/153 (81.5%) and 133/155 (85.5%); 9 mo: 145/153 (95.0%) and 151/155 (97.7%)	NR	Did not define, but presented no. of cases; 2 in each group (1.2%) were excluded after being retreated for presumptive symptomatic reinfection or relapse (ulcers or rash)	23
			Azithromycin (163)	57.7% of participants ≥25 y; 31.9; 51.5		Azithromycin 2.0 g by mouth, single dose		In 140 patients interviewed: nausea (8.6%, stomach pain (4.3%), diarrhea (0.7%), and vomiting (0.7%)			
Rolfs et al [26] (1997; USA) [Jan 1991–Jun 1994]	RCT	PCR/VDRL	BPG (276)	NR; NR; 15.2	Primary (total = 139); secondary (total = 253); early latent (total = 149)	Intramuscular BPG 2.4 MIU, single dose	Decrease in RPR titer by ≥2 dilutions or change to a nonreactive test result	At 3 mo: 135/175 (77.1%) and 139/185 (75.1%); 6 mo: 129/157 (82.2%) and 140/169 (82.8%); 9 mo: 125/153 (81.7%) and 124/148 (83.8%); 12 mo: 116/137 (84.7%) and 122/142 (86.0%)	NR	Defined and presented the no. of cases; 1 patient (0.2%) was considered reinfected (increase in RPR titer) on the basis of his sexual history	25
			BPG + amoxicillin + probenecid (265)	NR; NR; 22.3		Intramuscular BPG 2 400 000 IU (single dose) + oral amoxicillin 2.0 g (thrice daily for 10 d) + oral probenecid 500 mg (thrice daily for 10 d)					
Salado-Rasmussen et al [27] (2016; Copenhagen, Denmark) [May 2004–Oct 2009]	Retrospective cohort study	FTA-Abs, anti-flagellum IgM and IgG/RPR	BPG (75)	39 (Range, 24–61); 99; 100	Primary (8, 12); secondary (42, 75); early latent (10, 18); late latent (13, 21); relapse (0, 1); unknown (2, 0)	Intramuscular BPG 2.4 MIU , single dose, for early syphilis and 1 dose weekly for 3 wk for late latent syphilis	≥4-Fold decline in RPR titers after therapy	At 3 mo: 12/58 (20.7%) and 20/89 (22.5%;) 6 mo: 28/45 (62.2%) and 37/74 (50%); 12 mo: 40/48 (83.3%) and 66/78 (84.6%)	NR	NR	19
			Doxycycline (127)	40 (Range: 20–83); 99%; 100%		Doxycycline 100 mg by mouth, twice daily for 14 d for early syphilis (primary, secondary, early latent), and 30 d for late latent syphilis					
Shao et al [28] (2016; Tianjin, China) [Jan 2011–Dec 2013]	Retrospective cohort study	NR/RPR and VDRL	BPG (40)	67.5% of participants 20–39 y; 55.0; 0	Primary (23, 15, 14); secondary (17, 62, 65)*	Intramuscular BPG 2.4 MIU, single dose	VDRL or RPR titers became nonreactive after disappearance of clinical manifestations of syphilis within 2 y after treatment	Yes; 24 mo: 31 (77.5%), 56 (72.7%), and 69 (87.3%)	NR	NR	20
			Minocycline 2 wk (77)	70.1% of participants 20–39 y; 46.7; 0		Minocycline 100 mg by mouth, twice daily for 14 d					
			Minocycline 4 wk (79)	59.5% of participants 20–39 y; 44.3; 0		Minocycline 100 mg by mouth, twice daily for 28 d					
Smith et al [29] (2004; Texas, USA) [Jan 2007–Aug 2013]	RCT	MHA-TP/RPR and VDRL	Procaine penicillin G + probenecid (16)	35.4 (Range, 25–61); 81.2; 100	Late latent (16, 11); neurosyphilis (0, 4)	Intramuscular procaine penicillin G 2.4 MIU, once daily, with probenecid 500 mg by mouth, 4 times daily for 15 d	≥4-Fold decrease in RPR titer with no increase during time of observation	At 12 mo: 9/10 (90%) and 12/14 (85.7%); 24 mo: 7/10 (70%) and 6/14 (42.9%)	NR	Defined and presented the no. of cases: development of signs of a primary infection or neurological or clinical symptoms of syphilis during median follow-up period of 2 y; no patients found (0.0%)	19
			Ceftriaxone (15)	34.5 (Range, 23–56); 93.3; 100		Intramuscular ceftriaxone 1.0 g, once daily for 15 d					
Spornraft-Ragaller et al [30] (2011; Dresden, Germany) [Jan 2001–Dec 2008]	Retrospective cohort study	FTA-Abs, TPHA, TPPA, immunoblot/VDRL	Benzathine or clemizole penicillin G (12)	42 (Range: 33–57); 100%; 100%	Primary (2, 1); secondary (6, 6); early latent (3, 2); neurosyphilis (1, 2)	8 Patients received BPG 2.4 MIU intramuscular at weekly intervals for 3 wk (n = 7) or 2 wk (n = 1); 2 patients, intramuscular clemizole penicillin G 1 MIU daily for 14 or 21 d; and 2 patients intravenou penicillin G 10 MIU, thrice daily for 21 d	≥4-Fold decrease (or 2 dilutions) in VDRL titer or reversion of VDRL result to nonreactive	At 3 mo: 5/7 (71.4%) and 10/11 (90.9%); 12 mo: 11/11 (100%) and 11/12 (91.7%)	NR	Defined and presented no. of cases: considered when VDRL result rose ≥4-fold after previous reversion to negative (n = 2)	19
			Ceftriaxone (12)	40.5 (Range: 29–47); 100%; 100%		Intravenous ceftriaxone: 8 patients received 2.0 g, once daily for 10–14 d; 2 patients, 2.0 g for 21 d; and 2 patients, 1.0 g for 14 d					
Tsai et al [31] (2014; Taipei, Taiwan) [Jan 2007–Aug 2013]	Retrospective cohort study	TPHA/RPR	BPG (271)	31.4 (Range, 20–71); 10%; 100	Primary (24, 11); secondary (167, 51); early latent (80, 61)	Intramuscular BPG 2.4 MIU, single dose	≥4-Fold decline in RPR titer by 6 and 12 mo of treatment	At 6 mo: 196 (72.3%) and 78 (63.4%) [*P* = .08]; 12 mo: 185 (68.3%) and 81 (65.9%) [*P* = .68]	NR	Defined and presented no. of cases: new symptoms of primary or secondary syphilis or increase in RPR titer by ≥4-fold after ever achieving serological response during follow-up; penicillin group: 20% (15/20); doxycycline group: 6.7% (3/45)	20
			Doxycycline (123)	32.0 (Range, 20–59); 100; 100		Doxycycline 100 mg by mouth, twice daily for 14 d					
Wong et al [32] (2008; Alberta, Canada) [Jan 1980–Dec 2001]	Retrospective cohort study	MHA-TP and FTA-Abs/RPR	BPG (420)	43.6% of participants 20–29 y; 73.8; 0	100% Primary	Intramuscular BPG 2.4 MIU, single dose	≥4-Fold decrease in baseline RPR test antibody titer by 6 mo, 8-fold decrease by 12 mo, or 16-fold decrease by 24 m	409/420 (97.4%) and 25/25 (100%)	NR	NR	19
			Doxycycline or tetracycline (25)	48.0% of participants 20–29 y; 64.0; 0		Doxycycline 100 mg by mouth, twice daily for 14 d, or tetracycline 500 mg by mouth, 4 times daily for 14 d					
Wu et al [33] (2021; Tianjin, China) [Jan 2011–Dec 2017]	Retrospective cohort study	TPPA/RPR	BPG (118)	61.0% of participants <40 y; 59.3; 0	Primary (34, 39); secondary (45, 50); early latent (39, 69)	Intramuscular BPG 2.4 MIU, once weekly for 1 or 2 wk	Either negative RPR or ≥4-fold decrease after treatment	At 24 mo: 104 (81.3%) and 135 (85.4%) [*P* > .05]	NR	Did not define or present no. of cases; explicitly excluded reinfected patients from analysis (0.0%)	17
			Minocycline (158)	65.2% of participants <40 y; 51.3; 0		Minocycline 100 mg by mouth, twice daily for 28 d					
Xiao et al [34] (2017; Shandong, China) [Jan 2008–Dec 2014]	Retrospective cohort study	TPPA/RPR	BPG (496)	41.1% of participants 20–29 y; 49.2; 0	Primary (99, 19); secondary (252, 58); early latent (145, 28)	Intramuscular BPG 2.4 MIU, single dose	≥4-Fold decline in RPR titer from baseline value at 6 or 12 mo of treatment if initial RPR titer was ≥1:8; if RPR titer was 1:4, 1:2, or 1:1 at baseline for primary or secondary syphilis, treatment was considered successful when lesions disappeared and RPR turned negative after treatment	At 6 mo: 372 (75.0%) and 73 (69.5%) [*P* = .24]; 12 mo: 477 (96.2%) and 97 (92.4%) [*P* = .12]	NR	NR	19
			Doxycycline (105)	38.1% of participants 20–29 y; 40.0; 0		Doxycycline 100 mg by mouth, twice daily for 14 d					
Yang et al [35] 2016 (Taiwan) [Jan 2007–Apr 2014]	Prospective cohort study	TPPA and PCR/RPR	BPG (162)	32.0 (SD, 7.6); ≥99.4; 100	Primary (13, 33); secondary (82, 84); early latent (67, 120)	Intramuscular BPG 2.4 MIU, single dose	≥4-Fold decline in RPR titer by 12 mo after treatment	At 12 mo: 99 (61.1%) and 134 (56.5%) [*P* = .41]	NR	Defined and presented proportion of cases; reinfection was indicated by appearance of new chancres or ≥4-fold increases in RPR titers after initial achievement of serological response increased at 12-mo follow-up (56.3%)	21
			Azithromycin (237)	33.1 (SD, 7.6); ≥99.2; 100		Azithromycin 2.0 g by mouth, single dose			Diarrhea: 52.7%; nausea: 22.4%; abdominal pain: 18.6%; bloating: 17.7%; lassitude/somnolence: 27.4%		
Yuan et al [36] (2023; Hubei, China) [Mar 2020–Mar 2021]	Retrospective cohort study	NR/RPR and TRUST	BPG (32)	70.6 (SD, 5.7); 78.1; 0	100% Latent	Intramuscular BPG 2.4 MIU, once a week for 3–4 wk	Significantly effective: ≥4-fold decrease in serum RPR titer after treatment; effective: ≥2- to <4-fold decrease in serum RPR titers	Yes; 3 mo: 4 (12.5%) and 11 (28.9%) [*P* = .10]; 6 mo: 10 (31.2%) and 20 (55.3%) [*P* = .04]; 12 mo: 23 (71.9%) and 35 (92.1%) [*P* = .02]	Nausea: 6.2;, local pain: 3.1%; rash: 3.1%; vomiting: 0%; total: 12.5%	NR	20
			BPG + ceftriaxone (38)	71.0 (SD, 5.2); 65.8; 0		Intramuscular BPG 2.4 MIU, once weekly for 3–4 wk + intramuscular ceftriaxone 1.0 g, once daily for 10 d			Nausea: 2.6%; local pain: 5.3%; rash: 2.6%; vomiting: 5.3%; total: 15.8%		
Zengarini et al [37] (2022; Bologna, Italy) [Jan 2010–Jan 2020]	Retrospective cohort study	TPHA/RPR	BPG (41)	39.8 (SD, 13.4); 87.8; 43.9	Primary (7, 7); secondary (9, 9); early latent (8, 8); late latent (17, 17)	Intramuscular BPG 2.4 MIU, once weekly for 1–3 wk according to the stage (14 d for early syphilis, 28 d for other forms)	Negativization of RPR or ≥4-fold decrease in baseline RPR titer within 24 mo	At 12 mo: 26/36 (72.2%) and 22/33 (66.7%;) 24 mo: 37/37 (100%) and 27/31 (87.1%)	NR	Defined and presented no. of cases; emergence of new clinical signs and symptoms after treatment or a 4-fold increase in RPR during follow-up period was considered reinfection and excluded (0.0%)	17
			Doxycycline/tetracyclines (41)	40.5 (SD,11.1); 90.2; 41.5		Doxycycline 100 mg by mouth, twice daily for 14–28 d according to stage (14 d for early syphilis, 28 d for other forms)					

Abbreviations: BPG, benzathine penicillin G; CSF, cerebrospinal fluid; FTA-Abs, fluorescent treponemal antibody absorption; HIV, human immunodeficiency virus; Ig, immunoglobulin; IQR, interquartile range; ITT, intention-to-treat; J-H, Jarisch-Herxheimer; MHA-TP, microhemagglutination assay for *Treponema pallidum*; MIU, million international units; NR, not reported; PCR, polymerase chain reaction; RCT, randomized clinical trial; RPR, rapid plasma reagin; SD, standard deviation; TPHA, *T pallidum* hemagglutination assay; TPPA, *T pallidum* particle agglutination; TRUST, toluidine red unheated serum test.

*The first value refers to the control group (BPG), the second value refers to the first intervention group, and the third value refers to the second intervention group.

Of the 16 studies that mentioned reinfection [11–15, 17, 19, 20, 25, 26, 29–31, 33, 35, 37], 8 gave a definition for reinfection and presented the number of cases [11, 14, 26, 29–31, 35, 37], 4 did not define reinfection or give the number of reinfected patients [12, 15, 19, 20], and 4 did not define reinfection but presented the number of cases [13, 17, 25, 33]. Reinfection varied from no cases [17, 25, 29, 33, 37] to 56.3% [35] among the studies that provided the number or proportion of cases. Of the presented definitions of reinfection, the most common was a ≥4-fold increase of titers during follow-up period [11, 14, 30, 31, 35, 37]. Three studies added to this definition the development of new clinical symptoms [14, 31, 37]. Regarding quality assessment scores (Supplementary Table 3), 21 studies were considered of high quality (≥18 of the 28 possible points) [11–14, 16–20, 23, 25–32, 34–36] per the Downs and Black quality tool, and 6 were deemed fair (14–17 points) [15, 21, 22, 24, 33, 37].

We included a total of 6710 syphilis events from all 27 studies in our analysis. All studies in this systematic review included patients with nonneurological stages of syphilis. In addition, 4 included a total of 34 patients with neurosyphilis [15, 24, 29, 30]. Since the proportion of these patients was low (0.5% of all included patients), we opted to maintain these studies in our main analysis. However, we performed a stratified analysis of studies that did not include any participants with neurological syphilis (Supplementary Figures 1*H*–1*J*).

We intended to compare penicillin monotherapy with other strategies. As 4 studies considered 3 comparison groups each [18, 20, 24, 28], we had to choose a pair of groups to include in the meta-analysis. For Hook et al (2002) [18] and Shao et al [28], we chose the intervention group to be patients who received azithromycin (4 g) and minocycline (4 weeks), respectively, because the difference in outcomes was greater between these groups and the penicillin group. For Kiddugavu et al [20], and Psomas et al [24], the selected criteria were based on the similarity in the number of participants included in the penicillin and the intervention groups (azithromycin and ceftriaxone groups, respectively). For Hook et al (2010) [19], we included data reported in the intention-to-treat analysis because it maximized the number of included patients. On this first global analysis, the degree of heterogeneity was acceptable (*P* = .04; *I*^2^ = 36%). However, to mitigate this heterogeneity level and compare each treatment strategy more precisely, we performed a stratified analysis based on the comparator antibiotic regimen used.

**Figure 2.**
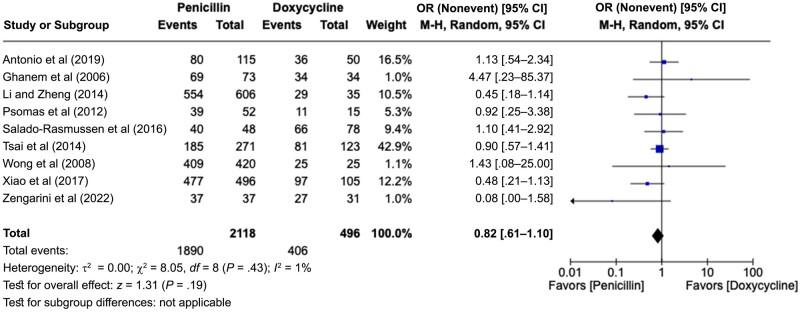
Forest plot of syphilis serological conversion after BPG monotherapy or doxycycline monotherapy [12, 17, 21, 24, 27, 31, 32, 34, 37]. Odds ratios (ORs) were determined using the Mantel-Haenszel random-effects method and are shown with 95% confidence intervals (CIs).

When analyzing the 9 studies that compared BPG with doxycycline (Figure 2) [12, 17, 21, 24, 27, 31, 32, 34, 37], we observed no significant differences between the rates of cure (pooled OR, 0.82 [95% CI, .61–1.10]) with low-heterogeneity results for the studies (*P* = .43; *I*^2^ = 1%). Expanding our analysis to studies that evaluated tetracyclines, we included 2 more about minocycline [28, 33] and performed a stratified analysis with 11 studies [12, 17, 21, 24, 27, 28, 31–34, 37], with similar conclusions (pooled OR, 0.87 [95% CI, .65–1.16]) and with low heterogeneity (*P* = .36; *I*^2^ = 9%).

**Figure 3.**
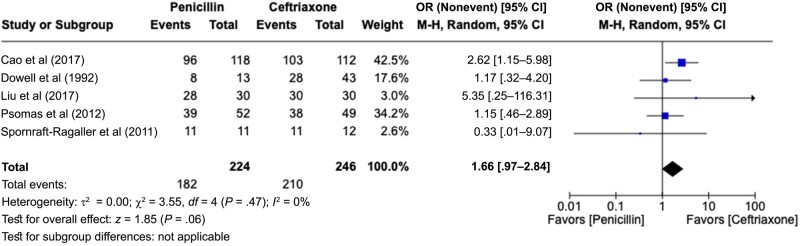
Forest plot of syphilis serological conversion after BPG monotherapy or ceftriaxone monotherapy [13, 15, 22, 24, 30]. Odds ratios (ORs) were determined with the Mantel-Haenszel random-effects method and are shown with 95% confidence intervals (CIs).

**Figure 4.**
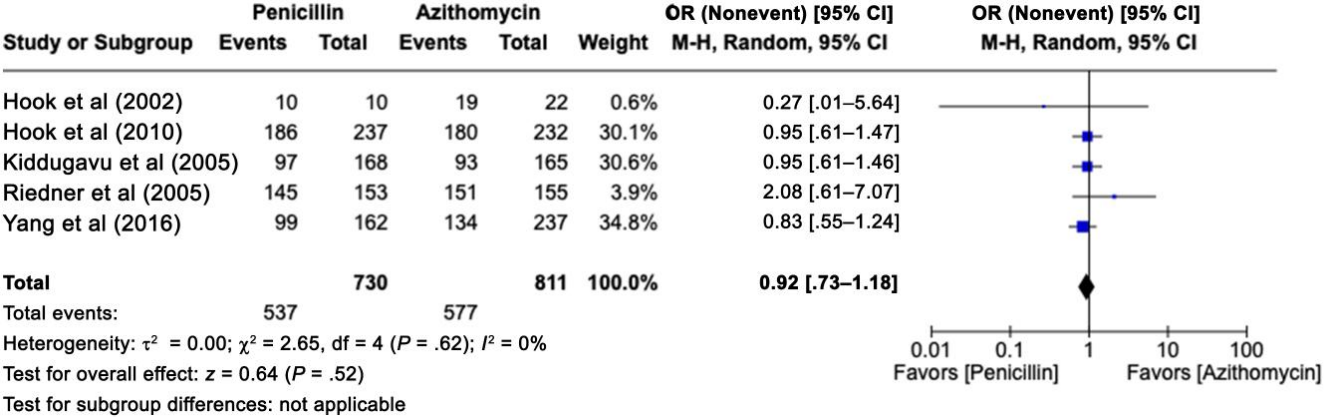
Forest plot of syphilis serological conversion after BPG monotherapy or azithromycin monotherapy [18–20, 25, 35]. Odds ratios (ORs) were determined with the Mantel-Haenszel random-effects method and are shown with 95% confidence intervals (CIs).

We also analyzed the 5 studies that compared BPG with ceftriaxone [13, 15, 22, 24, 30] (Figure 3). We observed no significant difference between the rates of cure (pooled OR, 1.66 [95% CI, .97–2.84]). This analysis was performed with low heterogeneity (*P* = .47; *I*^2^ = 0%). However, when we added the study that evaluated cefixime [23], another third-generation cephalosporin similar to ceftriaxone, the conclusion favored the use of cephalosporins (pooled OR, 1.67 [95% CI, 1.04–2.69]), with lower heterogeneity (*P* = .62; *I*^2^ = 0%). We included 5 studies comparing penicillin with azithromycin [18–20, 25, 35] (Figure 4) and found no difference in cure rates (pooled OR, 0.92 [95% CI, .73–1.18]), with homogeneous results (*P* = .62; *I*^2^ = 0%).

**Figure 5.**
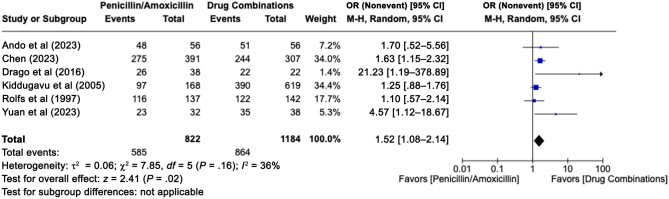
Forest plot of syphilis serological conversion after penicillin monotherapy or a strategy of treatment based on drug combinations [11, 14, 16, 20, 26, 36]. Odds ratios (ORs) were determined with the Mantel-Haenszel random-effects method and are shown with 95% confidence intervals (CIs).

Six studies compared penicillin with combination therapy [11, 14, 16, 20, 26, 36] (Figure 5). The 6 combined strategies found in the included studies were amoxicillin plus probenecid [11], penicillin plus doxycycline [14], penicillin plus ceftriaxone plus doxycycline [16], penicillin plus azithromycin [20], penicillin plus amoxicillin plus probenecid [26], and penicillin plus ceftriaxone [36]. The result was obtained with a low level of heterogeneity (*P* = .16; *I*^2^ = 36%) and showed a slight but significant difference in favor of the association strategies (pooled OR, 1.52 [95% CI, 1.08–2.14]).

The funnel plot (Supplementary Figure 2) revealed that the 27 studies included in the meta-analysis were reasonably balanced around the pooled ORs. Thus, there was little evidence of publication bias. The Egger test also did not indicate publication bias among those studies included in the meta-analysis (*P* = .81).

## DISCUSSION

In this systematic review and meta-analysis, various drug strategies have been shown as viable alternatives to penicillin in treating nonneurological syphilis. For example, monotherapy with drugs such as doxycycline, ceftriaxone, and azithromycin had outcomes similar to those of traditional penicillin monotherapy in terms of safety and efficacy. Combination therapies also appear to be viable alternatives.

To the best of our knowledge, the present study represents the most comprehensive systematic review and meta-analysis comparing multiple interventions for syphilis therapy, and it is the only review that includes studies evaluating drug combination strategies. Our results have shown that penicillin is not more effective than other strategies in terms of serological response. On the contrary, we have found similarities when comparing penicillin with tetracycline and azithromycin and a slight advantage favoring cephalosporins and combination treatment. These results are similar to those obtained by other reviews [38–40].

The acceptability of treatment to patients is a fundamental consideration in therapeutic recommendations. Understandably, patients may prefer oral or intramuscular administration over intravenous, and they may prefer taking medication as infrequently as possible, whether daily or weekly. Convenience is a crucial aspect of a treatment plan, as it significantly influences a patient's adherence to treatment until its completion. Given this, it might be more appropriate for some patients to take oral antibiotics (eg, doxycycline and azithromycin) instead of intravenous drugs (eg, ceftriaxone) or intramuscular drugs (eg, BPG). For instance, the potential benefits of shorter treatment durations, improved accessibility, and reduced costs [41] associated with alternative antibiotics could significantly enhance the feasibility and success of syphilis treatment, especially in settings with limited resources. Furthermore, coinfection with other bacteria, such as *Chlamydia trachomatis* and *Neisseria gonorrhoeae,* may not be uncommon, and patients could benefit from the use of doxycycline [42].

Globally, people with syphilis are commonly coinfected with HIV [43]. Our stratified analysis focusing exclusively on people living with HIV indicated that this population can derive equal benefits from alternative drug strategies with respect to efficacy and security. This knowledge holds significant relevance in the healthcare of these individuals, as they are at increased risk of becoming immunodeficient, necessitating careful consideration when selecting medications.

In their latest guidelines on treatment for sexually transmitted infections [5], the Centers for Disease Control and Prevention (CDC) recommends penicillin G as the first-line treatment for syphilis across all stages, but they do acknowledge alternative options for individuals with penicillin allergies. In this regard, the CDC exercises caution but endorses doxycycline and tetracycline as viable alternatives, with a preference for doxycycline, given its better compliance rates and reduced incidence of gastrointestinal adverse effects. Furthermore, the CDC notes the effectiveness of ceftriaxone and azithromycin but discourages the latter due to rising concerns about antibiotic resistance [44]. Our results do not invalidate penicillin as the reference standard for treating nonneurological syphilis. Instead, they indicate that there are consistent data affirming that other alternatives can be considered in scenarios where penicillin is not available.

Our study has several limitations. First, most studies (17 of 27) were nonrandomized. Second, there were missing data on important analyzed aspects, including adverse events related to drugs and information about reinfected patents. Therefore, we could not perform a statistical analysis focused on reinfection. In addition, variety concerning treponemal and nontreponemal tests may have affected the homogeneity of the analysis. Moreover, the absence of a standardized definition for serological cure and the varying criteria for the ideal serological cure time may have affected the homogeneity of our analysis. Heterogeneity was also observed in certain analyses, which could stem from variations in study designs, geographic locations, and patient populations.

Third, the discrepancy in the number of syphilis events across studies, ranging from 24 [30] to 952 [20], resulted in variations in the weighting of certain stratified analyses. In addition, a total of 34 patients with neurosyphilis (0.5% of the total sample size) could not be excluded for 4 included studies, all comparing penicillin with ceftriaxone. When we performed stratified analyses excluding all 4 studies that included patients with neurosyphilis, penicillin was not associated with improved serological cure rates. Discrepancies were also seen among drug regimens. The most compelling evidence to support further studies is based on the following regimens: intramuscular BPG weekly for 1–3 weeks [12–28, 30–37] or intramuscular procaine penicillin once daily for 15 days [29], depending on syphilis stage; oral doxycycline, ≥100 mg twice daily for 14–28 days [12, 17, 21, 27, 31, 32, 34, 37]; oral azithromycin, 1–2 g as a single dose [18–20, 25, 35]; and intravenous ceftriaxone, 1 g daily for 10 days [13, 22].

Fourth, it was beyond the scope of this review to assess the financial impact or other unintended consequences of adopting any of the analyzed drugs for syphilis treatment, especially in the context of population-wide strategies. Fifth, our inclusion criteria were limited to comparative studies, which required the presence of a control group (penicillin and derivatives) and ≥1 intervention group (involving other drugs or strategies). This approach excluded studies that focused solely on a single drug, potentially limiting our understanding of adverse effects and associated costs for that specific drug. Sixth, our review specifically excluded studies focused on children, neurosyphilis, ocular syphilis, and otosyphilis. Therefore, it remains uncertain whether the conclusions drawn from our meta-analysis can be extrapolated to patients with these conditions. It is vital to note that while our study focused on early syphilis stages, it is particularly important for healthcare providers to conduct a thorough workup to ensure the proper staging of syphilis, as inadequate treatment in more advanced cases can lead to poor outcomes. Seventh, none of the drug combination strategies were evaluated by more than one study, which has limited our stratified analysis. We were only able to evaluate a “drug combination” group, without stratifying it into specific strategies.

In conclusion, considering our findings and limitations, it is evident that further research is essential in syphilis therapy. More randomized controlled trials are needed to establish a robust foundation for treatment recommendations. In addition, the absence of standardized definitions for serological cure and ideal time frames for seroconversion necessitates the development of uniform criteria to enhance the consistency of future analyses. Moreover, extending investigations to include patients with neurosyphilis, ocular syphilis, and otosyphilis is crucial, given our study's focus on nonneurological syphilis. Finally, studying the financial impacts of various treatment options is crucial as population-wide syphilis management strategies advance. Our study indicates that alternative drug strategies, such as ceftriaxone, azithromycin, and doxycycline monotherapies, can reduce the dependency on penicillin for nonneurological syphilis, including among HIV-positive patients, in scenarios where penicillin use is not feasible.

## Supplementary Material

ofae142_Supplementary_Data

## Supplementary Data

Supplementary materials are available at *Open Forum Infectious Diseases* online. Consisting of data provided by the authors to benefit the reader, the posted materials are not copyedited and are the sole responsibility of the authors, so questions or comments should be addressed to the corresponding author.
