# Supplementary material for: Syphilis Treatment: Systematic Review and Meta-Analysis Investigating Nonpenicillin Therapeutic Strategies
Source: Open Forum Infect Dis. 2024 Mar 13;11(4):ofae142. doi: 10.1093/ofid/ofae142 (PMC11002953; doi:10.1093/ofid/ofae142)
Supplement: ofae142_Supplementary_Data [file ofae142_supplementary_data.docx]

**Supplemental Table 1**: PubMed search strategy

| Group 1 | Group 2 | Group 3 |
| --- | --- | --- |
| Treatment  OR  Antibiotic(s)  OR  Anti-Bacterial Agents  OR  Antitreponemal Agents | Syphilis  OR  Treponema | Amoxicillin  OR  Ampicillin  OR  Azithromycin  OR  Cefazolin  OR  Cefixime  OR  Ceftriaxone  OR  Cephalosporins  OR  Chloramphenicol  OR  Doxycycline  OR  Ethylenediamines  OR  Erythromycin  OR  Fleroxacin  OR  Immunoglobulin  OR  Linezolid  OR  Macrolides  OR  Metronidazole  OR  Minocycline  OR  Prednisone  OR  Probenecid  OR  Propylamines  OR  Spectinomycin  OR  Sulfamethoxazole  OR  Tinidazole  OR  Trimethoprim  OR  Tetracycline |

(((((((("treatment"[Title/Abstract]) OR ("antibiotic"[Title/Abstract])) OR ("antibiotics"[Title/Abstract])) OR ("anti bacterial agents"[Title/Abstract])) OR ("antitreponemal"[Title/Abstract])) AND (("syphilis"[Title/Abstract]) OR ("treponema"[Title/Abstract]))) AND ((((((((((((((((((((((((("amoxicillin"[Title/Abstract]) OR ("ampicillin"[Title/Abstract])) OR ("azithromycin"[Title/Abstract])) OR ("cefazolin"[Title/Abstract])) OR ("cefixime"[Title/Abstract])) OR ("ceftriaxone"[Title/Abstract])) OR ("cephalosporins"[Title/Abstract])) OR ("chloramphenicol"[Title/Abstract])) OR ("doxycycline"[Title/Abstract])) OR ("ethylenediamines"[Title/Abstract])) OR ("erythromycin"[Title/Abstract])) OR ("fleroxacin"[Title/Abstract])) OR ("immunoglobulin"[Title/Abstract])) OR ("linezolid"[Title/Abstract])) OR ("macrolides"[Title/Abstract])) OR ("metronidazole"[Title/Abstract])) OR ("minocycline"[Title/Abstract])) OR ("prednisone"[Title/Abstract])) OR ("probenecid"[Title/Abstract])) OR ("propylamines"[Title/Abstract])) OR ("spectinomycin"[Title/Abstract])) OR ("sulfamethoxazole"[Title/Abstract])) OR ("tinidazole"[Title/Abstract])) OR ("trimethoprim"[Title/Abstract])) OR ("tetracycline"[Title/Abstract]))) OR ((((((("treatment"[Text Word]) OR ("antibiotic"[Text Word])) OR ("antibiotics"[Text Word])) OR ("anti bacterial agents"[Text Word])) OR ("antitreponemal"[Text Word])) AND (("syphilis"[Text Word]) OR ("treponema"[Text Word]))) AND ((((((((((((((((((((((((("amoxicillin"[Text Word]) OR ("ampicillin"[Text Word])) OR ("azithromycin"[Text Word])) OR ("cefazolin"[Text Word])) OR ("cefixime"[Text Word])) OR ("ceftriaxone"[Text Word])) OR ("cephalosporins"[Text Word])) OR ("chloramphenicol"[Text Word])) OR ("doxycycline"[Text Word])) OR ("ethylenediamines"[Text Word])) OR ("erythromycin"[Text Word])) OR ("fleroxacin"[Text Word])) OR ("immunoglobulin"[Text Word])) OR ("linezolid"[Text Word])) OR ("macrolides"[Text Word])) OR ("metronidazole"[Text Word])) OR ("minocycline"[Text Word])) OR ("prednisone"[Text Word])) OR ("probenecid"[Text Word])) OR ("propylamines"[Text Word])) OR ("spectinomycin"[Text Word])) OR ("sulfamethoxazole"[Text Word])) OR ("tinidazole"[Text Word])) OR ("trimethoprim"[Text Word])) OR ("tetracycline"[Text Word])))) OR (((("anti bacterial agents"[MeSH Terms]) OR ("antitreponemal agents"[MeSH Terms])) AND (("syphilis"[MeSH Terms]) OR ("treponema"[MeSH Terms]))) AND ((((((((((((((((((((((((("amoxicillin"[MeSH Terms]) OR ("ampicillin"[MeSH Terms])) OR ("azithromycin"[MeSH Terms])) OR ("cefazolin"[MeSH Terms])) OR ("cefixime"[MeSH Terms])) OR ("ceftriaxone"[MeSH Terms])) OR ("cephalosporins"[MeSH Terms])) OR ("chloramphenicol"[MeSH Terms])) OR ("doxycycline"[MeSH Terms])) OR ("ethylenediamines"[MeSH Terms])) OR ("erythromycin"[MeSH Terms])) OR ("fleroxacin"[MeSH Terms])) OR ("immunoglobulins"[MeSH Terms])) OR ("linezolid"[MeSH Terms])) OR ("macrolides"[MeSH Terms])) OR ("metronidazole"[MeSH Terms])) OR ("minocycline"[MeSH Terms])) OR ("prednisone"[MeSH Terms])) OR ("probenecid"[MeSH Terms])) OR ("propylamines"[MeSH Terms])) OR ("spectinomycin"[MeSH Terms])) OR ("sulfamethoxazole"[MeSH Terms])) OR ("tinidazole"[MeSH Terms])) OR ("trimethoprim"[MeSH Terms])) OR ("tetracycline"[MeSH Terms])))

**Supplemental Table 2**: Results of stratified analyses

| Study | Analysis | N of studies | Pooled OR (95%CI) | P value | Heterogeneity | |
| --- | --- | --- | --- | --- | --- | --- |
|  |  |  |  |  | I^2^ (%) | P value |
| Serological conversion | All studies | 27 | 1.13 (0.92-1.40) | 0.23 | 36 | <0.001 |
|  | Studies published in 2013-2023 | 16 | 1.18 (0.86-1.62) | 0.30 | 56 | 0.003 |
|  | Studies published in before 2013 | 11 | 1.12 (0.89-1.41) | 0.34 | 0 | 0.80 |
|  | Randomized clinical trials | 10 | 1.41 (0.91-2.18) | 0.12 | 36 | 0.12 |
|  | Cohort studies | 17 | 1.05 (0.83-1.34) | 0.69 | 38 | 0.06 |
|  | Location: North America | 7 | 0.97 (0.69-1.36) | 0.86 | 0 | 0.73 |
|  | Location: Europe | 5 | 1.06 (0.36-3.08) | 0.91 | 48 | 0.11 |
|  | Location: Asia | 12 | 1.19 (0.84-1.67) | 0.33 | 60 | 0.004 |
|  | Location: Africa | 2 | 1.30 (0.93-1.81) | 0.13 | 0 | 0.43 |
|  | Treponemal test: only TPHA | 4 | 1.07 (0.71-1.63) | 0.74 | 37 | 0.19 |
|  | Treponemal test: only TPPA | 5 | 1.24 (0.73-2.13) | 0.42 | 65 | 0.02 |
|  | Treponemal test: only FTA-Abs | 3 | 0.96 (0.62-1.47) | 0.84 | 0 | 0.42 |
|  | Non-treponemal test: only RPR | 15 | 1.02 (0.77-1.35) | 0.90 | 47 | 0.02 |
|  | Non-treponemal test: onlyVDRL | 5 | 1.19 (0.73-1.95) | 0.49 | 15 | 0.32 |
|  | Non-treponemal test: only TRUST | 3 | 1.03 (0.53 (1.98) | 0.94 | 57 | 0.10 |
|  | Only HIV positive patients | 7 | 0.94 (0.73-1.21) | 0.62 | 0 | 0.90 |
|  | Only HIV negative patients | 10 | 1.19 (0.76-1.88) | 0.45 | 55 | 0.02 |
|  | Both HIV positive and HIV negative patients | 10 | 1.29 (0.91-1.83) | 0.16 | 34 | 0.14 |
|  | Studies that did not include neurosyphilis patients | 23 | 1.16 (0.93-1.45) | 0.18 | 42 | 0.02 |
|  | Studies that included patients with and without neurosyphilis | 4 | 0.90 (0.46-1.77) | 0.77 | 0 | 0.54 |
|  | Serological cure definition: ≥4-fold (or 2 dilutions) decline in titers of the nontreponemal test adopted | 24 | 1.10 (0.89-1.38) | 0.38 | 40 | 0.02 |
|  | Serological cure definition: ≥4-fold (or 2 dilutions) decline in titers of the nontreponemal test adopted in 12 months | 4 | 1.29 (0.77-2.17) | 0.34 | 58 | 0.07 |
|  | Serological cure definition: ≥4-fold (or 2 dilutions) decline in titers of the nontreponemal test adopted in 6 months | 6 | 1.27 (0.62-2.58) | 0.51 | 61 | 0.03 |
|  | Studies that reported drug adverse effects | 9 | 1.54 (0.96-2.45) | 0.07 | 55 | 0.02 |
|  | Studies that defined reinfection and presented the number of cases | 9 | 1.10 (0.78-1.56) | 0.59 | 46 | 0.06 |
|  | High quality studies (D&B ≥18) | 21 | 1.21 (0.97-1.51) | 0.09 | 36 | 0.05 |

**Supplemental Table 3:** Details of Downs and Black (D&B) scale for each study

| Study | Reporting (Max 11 points) | External validity (Max 3 points) | Internal validity – bias (Max 7 points) | Internal validity – confounding (selection bias) (Max 6 points) | Power (Max 1 point) | Total score |
| --- | --- | --- | --- | --- | --- | --- |
| Ando, 2023 | 10 | 3 | 5 | 6 | 1 | 25 |
| Antonio, 2019 | 9 | 3 | 5 | 3 | 0 | 20 |
| Cao, 2017 | 8 | 3 | 5 | 5 | 0 | 21 |
| Chen, 2023 | 10 | 2 | 5 | 4 | 0 | 21 |
| Dowell, 1992 | 7 | 3 | 4 | 3 | 0 | 17 |
| Drago, 2016 | 11 | 1 | 5 | 3 | 0 | 20 |
| Ghanem, 2006 | 8 | 3 | 4 | 3 | 0 | 18 |
| Hook, 2002 | 10 | 3 | 3 | 5 | 0 | 21 |
| Hook, 2010 | 10 | 3 | 4 | 5 | 0 | 22 |
| Kiddugavu, 2005 | 8 | 3 | 4 | 3 | 0 | 18 |
| Li, 2014 | 9 | 3 | 3 | 1 | 0 | 16 |
| Liu, 2017 | 7 | 1 | 5 | 3 | 0 | 16 |
| Pei, 2021 | 10 | 2 | 5 | 5 | 0 | 22 |
| Psomas, 2012 | 7 | 3 | 4 | 2 | 0 | 16 |
| Riedner, 2005 | 10 | 3 | 5 | 5 | 0 | 23 |
| Rolfs, 1997 | 9 | 3 | 7 | 5 | 1 | 25 |
| Salado-Rasmussen, 2016 | 9 | 3 | 4 | 3 | 0 | 19 |
| Shao, 2016 | 10 | 3 | 4 | 3 | 0 | 20 |
| Smith, 2004 | 9 | 3 | 4 | 3 | 0 | 19 |
| Spornraft-Ragaller, 2011 | 9 | 3 | 5 | 2 | 0 | 19 |
| Tsai, 2014 | 10 | 3 | 5 | 2 | 0 | 20 |
| Wong, 2008 | 9 | 3 | 3 | 4 | 0 | 19 |
| Wu, 2021 | 8 | 3 | 4 | 2 | 0 | 17 |
| Xiao, 2017 | 9 | 3 | 5 | 2 | 0 | 19 |
| Yang, 2015 | 10 | 3 | 5 | 3 | 0 | 21 |
| Yuan, 2023 | 11 | 2 | 5 | 2 | 0 | 20 |
| Zengarini, 2022 | 9 | 2 | 3 | 3 | 0 | 17 |

**Supplemental Figure 1**: Forest plots

**A: All studies**


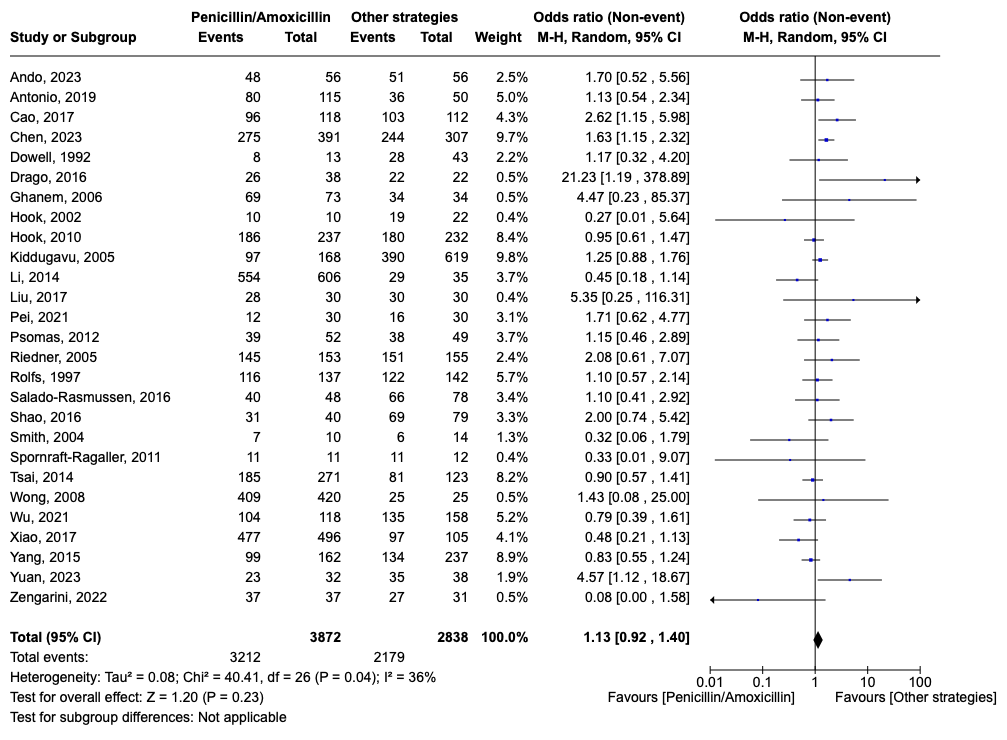


**B: RCT studies**


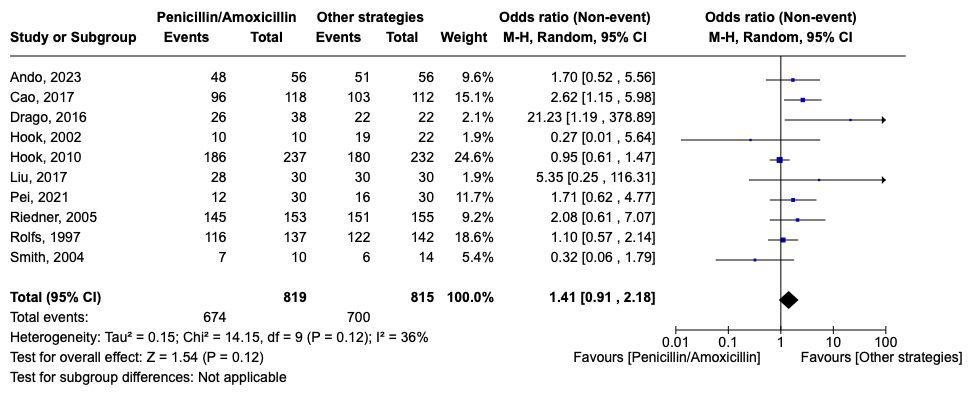


**C: Only HIV positive patients**


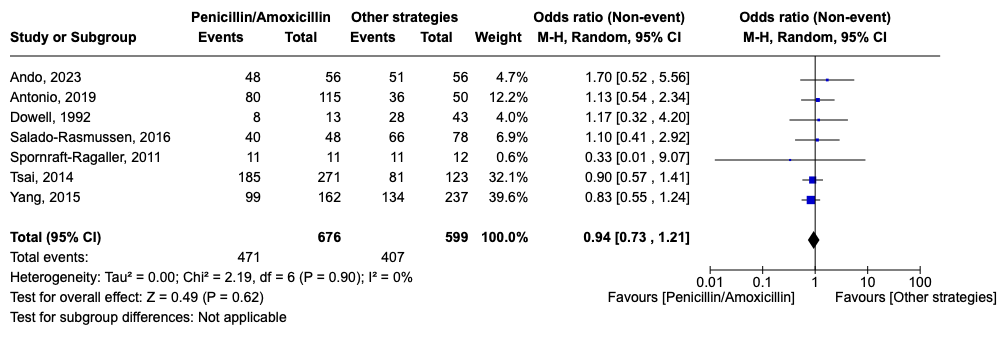


**D: Only HIV negative patients**


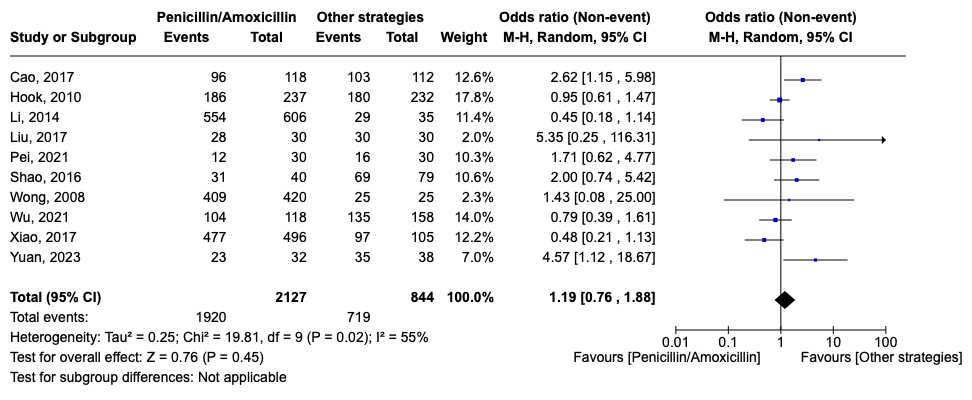


**E: Both HIV positive and HIV negative patients**

**
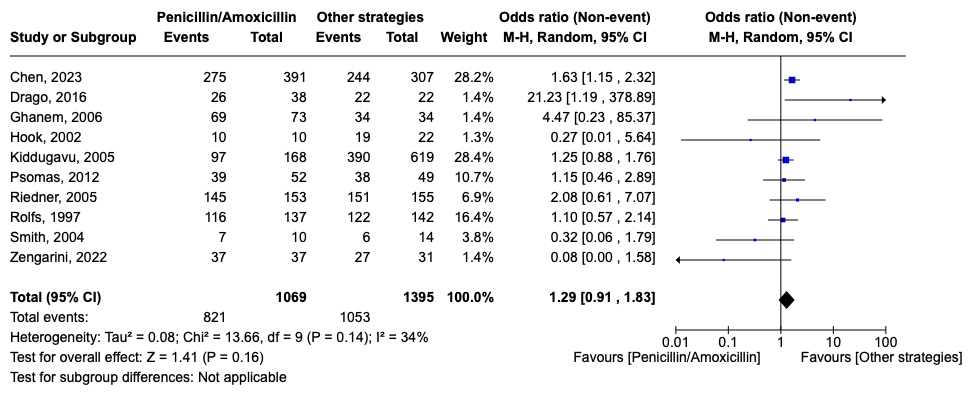
**

**F: Benzathine penicillin monotherapy vs. Cephalosporin monotherapy**

**
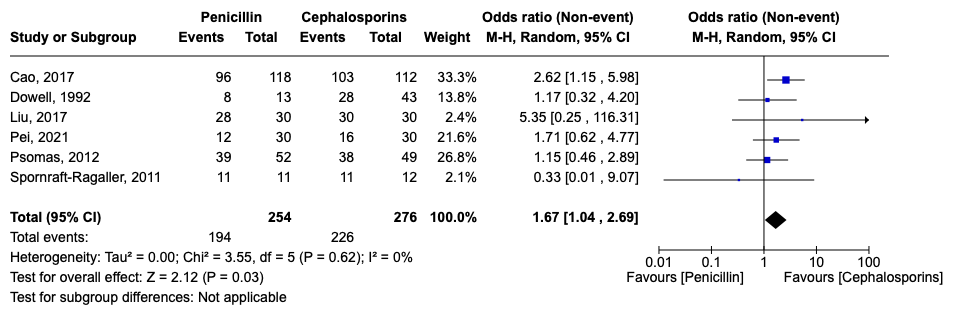
**

**G: Benzathine penicillin monotherapy vs. Tetracycline monotherapy**


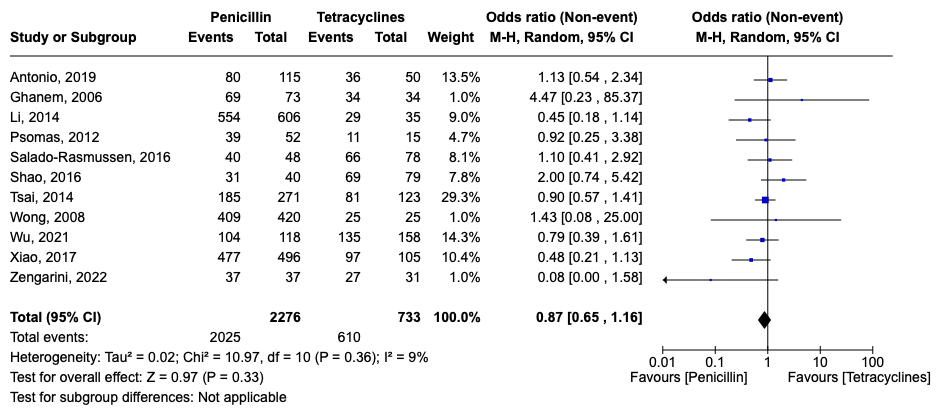


**H: Studies that did not include any participants with neurological syphilis**

**
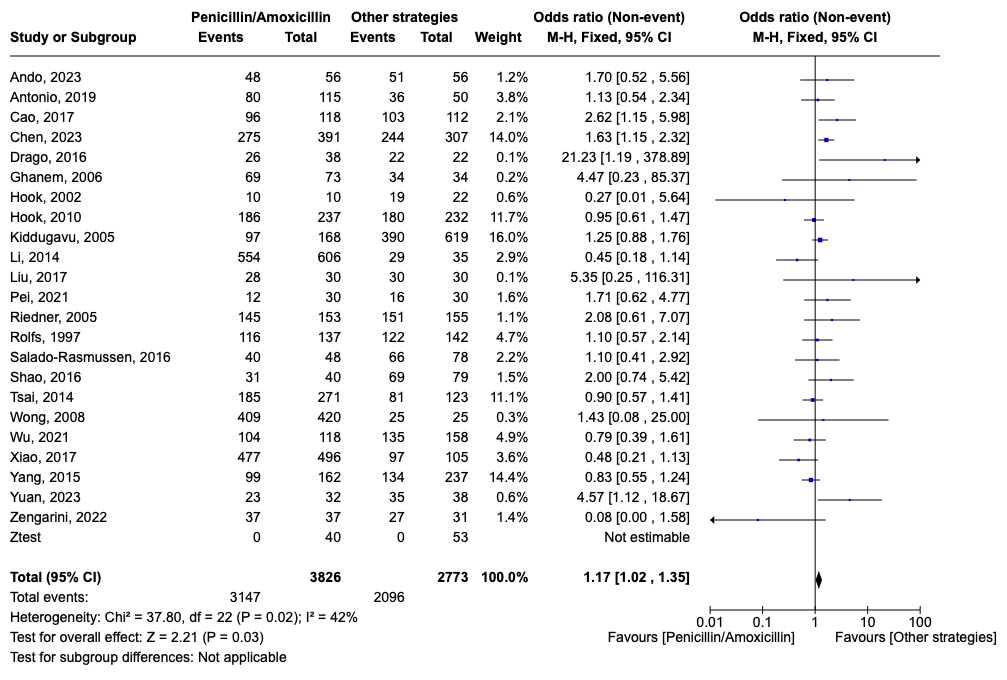
**

**I: Benzathine penicillin monotherapy vs. Ceftriaxone monotherapy excluding studies with neurosyphilis patients**

**
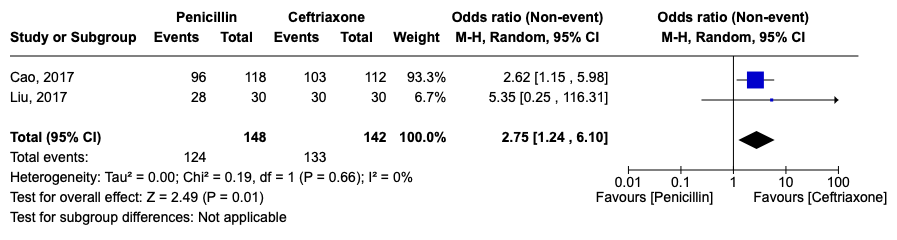
**

**J: Benzathine penicillin monotherapy vs. Cephalosporin monotherapy excluding studies with neurosyphilis patients**

**
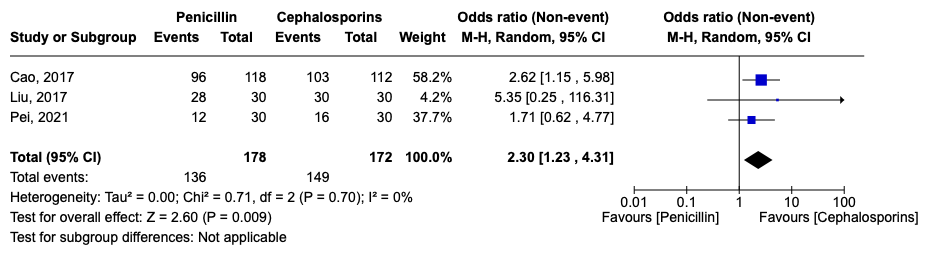
**

**Supplemental Figure 2**: Funnel plot

SE: Standard Error, OR: Odds Ratio


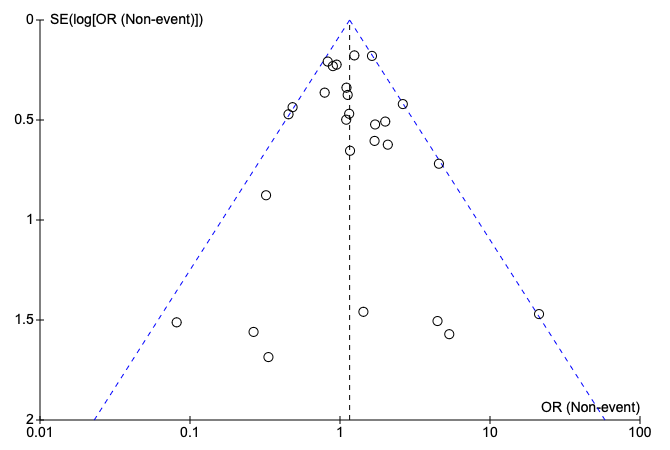


**Supplemental Form 1**: Data Abstract Form

1. **Journal:** ____________________________________
2. **First author last name**: __________
3. **Publication year**: __________
4. **Study location (city, state and country)**: __________
5. **Reviewer's initials**: __________

**Part 1**. Basic inclusion/exclusion criteria

1. Did the study evaluate **compare two or more drugs for the treatment of syphilis**?
   1. Yes
   2. No (If no, exclude it)
2. Which were the analyzed drugs in the **control group**?
   1. Penicillin G
   2. Amoxicillin
   3. Other: _______________________________
3. Which were the analyzed drugs in the **intervention group(s)**?
   1. Doxycycline
   2. Ceftriaxone
   3. Azithromycin
   4. Minocycline
   5. Amoxicillin
   6. Amoxicillin + Probenecid
   7. Cefixime
   8. Other: __________
4. What was the **nontreponemal (a) and treponemal (b) test** used? Feel free to highlight more than one.
   1. Nontreponemal antigen test:
      1. RPR
      2. VDRL
      3. Other: __________
   2. Treponemal test:
      1. FTA-Abs
      2. Other: __________
5. Does the study include HIV positive patients?
   1. Yes, the analysis is limited to this population
   2. Yes, but HIV negative participants were also included
   3. No
6. Does the study include patients with neurosyphilis/otosyphilis/ocular syphilis?
   1. Yes
   2. No
7. Duration of the study (length of time from the start of the research or clinical trial until its completion) in months: __________
   1. Initial and final month/year (e.g.: Feb/2006–Nov/2008): _________________
8. Our study aims to comprehensively analyze the efficacy of alternative treatments for syphilis, moving beyond the conventional use of penicillin. Knowing that, do you believe this study should be **excluded**?

(For example, if the study does not compare two different treatments for non-neurologic syphilis, it should be excluded)

- 1. Yes
  2. No

1. If "Yes", why? __________________________________________________

**Part 2**. Exposure and outcomes assessment (**check more than one if necessary**)

1. Where did the study take place?
   1. An academic medical center
   2. A community hospital
   3. Other: __________
2. Which type is this **study design**?
   1. Retrospective cohort study
   2. Prospective cohort study
   3. Case-control study
   4. Randomized controlled trial
   5. Other: __________
3. If the study was **randomized**: did it perform intention-to-treat or per protocol analyses?
   1. Yes:
      1. Intention-to-treat
      2. Per protocol
   2. No
4. Was the study performed in more than one hospital?
   1. Yes (please write the number of hospitals): _____
   2. No
5. What were the **dosage and duration** of treatment offered for the **intervention** group? (If there are more than one intervention group, please specify)

(e.g.: Ceftriaxone 2.0 g i.v. once daily for 10 days)

__________________________________________________________________________________________________________________________________________________

1. What were the **dosage and duration** of treatment offered for the **control** group?

____________________________________________________________________________________________________________________________________________________________________________________________________________________________________________________________________________________________________

1. Through which **parameters** does the study compare the control and the intervention groups?
   1. 3-month response rate
   2. 6-month response rate
   3. 12-month response rate
   4. 24-month response rate
   5. Relapse rate (recurrence of the disease's symptoms after an initial period of improvement or apparent recovery)
   6. Serofast rate (a less than four-fold decrease in non-treponemal antibody titles during a 6-month follow-up period in the absence of symptoms of syphilis or ≥2-Dilution Decrease in RPR Titer)
   7. Cure rate (a ≥4-fold decline following therapy or ≥2-Dilution Decrease in RPR Titer)
   8. Failure rate (a lack of a 4-fold decline or ≥2-Dilution Decrease in RPR Titer)
   9. Others: __________________________________________________________________________________________________________________________________________________________________________________________
2. Does the study explore adverse drug events?
   1. No
   2. Yes (please describe the potential adverse effects associated with the studied drugs):
      1. Control drug: ________________________________________________________________________________________________________________________________________________________________________
      2. Intervention drug: ________________________________________________________________________________________________________________________________________________________________________
3. Was there difference between the control and the intervention groups? In which outcomes? _________________________________________________________________________________________________________________________________________________________________________________________________________
4. Population characteristics:

|  | Control group | Intervention group | Intervention group 2 (if necessary) |
| --- | --- | --- | --- |
| Number of participants |  |  |  |
| Age (Mean [SD] or Median [IQR]) |  |  |  |
| % of female |  |  |  |
| Number of pregnant women |  |  |  |
| Number of HIV positive participants |  |  |  |
| Number of patients with neurosyphilis |  |  |  |
| Number of patients with otosyphilis |  |  |  |
| Number of patients with ocular syphilis |  |  |  |
| Number of patients in each stage of syphilis | Primary: __  Secondary: __  Latent: __  Tertiary: __ | Primary: __  Secondary: __  Latent: __  Tertiary: __ | Primary: __  Secondary: __  Latent: __  Tertiary: __ |

**Part 3**. Unadjusted and adjusted associations

1. Raw numbers: Please fill raw data for the following tables if available. Only include measures of effect if they are listed in the article.

Tables: Main association of interest - Positive cultures (total number)

| **3-month response** | Control group | Intervention group | Intervention group 2 |
| --- | --- | --- | --- |
| Cure |  |  |  |
| Failure |  |  |  |

Obs.: Serological cure is commonly defined as a ≥4-fold decline following therapy.

Serological failure is commonly defined as a lack of a 4-fold decline.

| **6-month response** | Control group | Intervention group | Intervention group 2 |
| --- | --- | --- | --- |
| Cure |  |  |  |
| Failure |  |  |  |

| **12-month response** | Control group | Intervention group | Intervention group 2 |
| --- | --- | --- | --- |
| Cure |  |  |  |
| Failure |  |  |  |

| **24-month response** | Control group | Intervention group | Intervention group 2 |
| --- | --- | --- | --- |
| Cure |  |  |  |
| Failure |  |  |  |

| **Cure** | Control group | Intervention group | Intervention group 2 |
| --- | --- | --- | --- |
| Yes |  |  |  |
| No |  |  |  |

How was “cure” defined? ____________________________________________________ __________________________________________________________________________________________________________________________________________________

| **Failure** | Control group | Intervention group | Intervention group 2 |
| --- | --- | --- | --- |
| Yes |  |  |  |
| No |  |  |  |

How was “failure” defined? ____________________________________________________ __________________________________________________________________________________________________________________________________________________

| **Serofast** | Control group | Intervention group | Intervention group 2 |
| --- | --- | --- | --- |
| Yes |  |  |  |
| No |  |  |  |

How was “serofast” defined? __________________________________________________ __________________________________________________________________________________________________________________________________________________

| **Relapse** | Control group | Intervention group | Intervention group 2 |
| --- | --- | --- | --- |
| Yes |  |  |  |
| No |  |  |  |

How was “relapse” defined? __________________________________________________ __________________________________________________________________________________________________________________________________________________

| **Other outcome (please complete)** | Control group | Intervention group | Intervention group 2 |
| --- | --- | --- | --- |
| Yes |  |  |  |
| No |  |  |  |

| **Other outcome (please complete)** | Control group | Intervention group | Intervention group 2 |
| --- | --- | --- | --- |
| Yes |  |  |  |
| No |  |  |  |

**Part 4**. Other references

Please look through the references. Are there other references that we should evaluate for the meta-analysis? If yes, please provide first author, journal and year:

________________________________________________________________________________________________________________________________________________________________________________________________________________________

**Part 5**. Quality Assessment Tool

Adapted Downs and Black Tool:

1. Is the hypothesis/aim/objective of the study clearly described?

| Yes | 1 |
| --- | --- |
| No | 0 |

1. Are the main outcomes to be measured clearly described in the Introduction or Methods section?
   1. If the main outcomes are first mentioned in the Results section, the question should be answered no.

| Yes | 1 |
| --- | --- |
| No | 0 |

1. Are the characteristics of the participants included in the study clearly described?
   1. In cohort and cross-sectional studies, inclusion and/or exclusion criteria should be given. In case-control studies, a case-definition and the sources for controls should be given.

| Yes | 1 |
| --- | --- |
| No | 0 |

1. Are the interventions of interest clearly described?
   1. Treatments and placebo (where relevant) that are to be compared should be clearly described.

| Yes | 1 |
| --- | --- |
| No | 0 |

1. Are the distributions of principal confounders in each group of subjects to be compared clearly described?
   1. A list of principal confounders is provided.

| Yes | 2 |
| --- | --- |
| Partially | 1 |
| No | 0 |

1. *Are the main findings of the study clearly described?*
   1. Simple outcome data (including denominators and numerators) should be reported for all major findings so that the reader can check the major analyses and conclusions. (This question does not cover statistical tests which are considered below).

| Yes | 1 |
| --- | --- |
| No | 0 |

1. *Does the study provide estimates of the random variability in the data for the main outcomes?*
   1. In non-normally distributed data the interquartile range of results should be reported. In normally distributed data the standard error, standard deviation or confidence intervals should be reported. If the distribution of the data is not described, it must be assumed that the estimates used were appropriate and the question should be answered yes.

| Yes | 1 |
| --- | --- |
| No | 0 |

1. *Have all important adverse events that may be a consequence of the intervention been reported?*
   1. This should be answered yes if the study demonstrates that there was a comprehensive attempt to measure adverse events. (A list of possible adverse events is provided).

| Yes | 1 |
| --- | --- |
| No | 0 |

1. *Have the characteristics of patients lost to follow-up been described?*
   1. This should be answered yes where there were no losses to follow-up or where losses to follow-up were so small that findings would be unaffected by their inclusion. This should be answered no where a study does not report the number of patients lost to follow-up.

| Yes | 1 |
| --- | --- |
| No | 0 |

1. *Have actual probability values been reported (e.g. 0.035 rather than <0.05) for the main outcomes except where the probability value is less than 0.001?*

| Yes | 1 |
| --- | --- |
| No | 0 |

*External validity:*

*All the following criteria attempt to address the representativeness of the findings of the study and whether they may be generalized to the population from which the study subjects were derived.*

1. *Were the subjects asked to participate in the study representative of the entire population from which they were recruited?*
   1. The study must identify the source population for patients and describe how the patients were selected. Patients would be representative if they comprised the entire source population, an unselected sample of consecutive patients, or a random sample. Random sampling is only feasible where a list of all members of the relevant population exists. Where a study does not report the proportion of the source population from which the patients are derived, the question should be answered as unable to determine.

| Yes | 1 |
| --- | --- |
| No | 0 |
| Unable to determine | 0 |

1. *Were those subjects who were prepared to participate representative of the entire population from which they were recruited?*
   1. The proportion of those asked who agreed should be stated. Validation that the sample was representative would include demonstrating that the distribution of the main confounding factors was the same in the study sample and the source population.

| Yes | 1 |
| --- | --- |
| No | 0 |
| Unable to determine | 0 |

1. *Were the staff, places, and facilities where the patients were treated, representative of the treatment the majority of patients receive?*
   1. For the question to be answered yes the study should demonstrate that the intervention was representative of that in use in the source population. The question should be answered no if, for example, the intervention was undertaken in a specialist center unrepresentative of the hospitals most of the source population would attend.

| Yes | 1 |
| --- | --- |
| No | 0 |
| Unable to determine | 0 |

*Internal validity – bias*

1. *Was an attempt made to blind study subjects to the intervention they have received ?*
   1. For studies where the patients would have no way of knowing which intervention they received, this should be answered yes.

| Yes | 1 |
| --- | --- |
| No | 0 |
| Unable to determine | 0 |

1. *Was an attempt made to blind those measuring the main outcomes of the intervention?*

| Yes | 1 |
| --- | --- |
| No | 0 |
| Unable to determine | 0 |

1. *If any of the results of the study were based on “data dredging”, was this made clear?*
   1. Any analyses that had not been planned at the outset of the study should be clearly indicated. If no retrospective unplanned subgroup analyses were reported, then answer yes.

| Yes | 1 |
| --- | --- |
| No | 0 |
| Unable to determine | 0 |

1. *In trials and cohort studies, do the analyses adjust for different lengths of follow-up of patients, or in case-control studies, is the time period between the intervention and outcome the same for cases and controls ?*
   1. Where follow-up was the same for all study patients the answer should be yes. If different lengths of follow-up were adjusted for by, for example, survival analysis the answer should be yes. Studies where differences in follow-up are ignored should be answered no.

| Yes | 1 |
| --- | --- |
| No | 0 |
| Unable to determine | 0 |

1. *Were the statistical tests used to assess the main outcomes appropriate?*
   1. The statistical techniques used must be appropriate to the data. For example non- parametric methods should be used for small sample sizes. Where little statistical analysis has been undertaken but where there is no evidence of bias, the question should be answered yes. If the distribution of the data (normal or not) is not described it must be assumed that the estimates used were appropriate and the question should be answered yes.

| Yes | 1 |
| --- | --- |
| No | 0 |
| Unable to determine | 0 |

1. *Was compliance with the intervention/s reliable?* Where there was non compliance with the allocated treatment or where there was contamination of one group, the question should be answered no. For studies where the effect of any misclassification was likely to bias any association to the null, the question should be answered yes.

| Yes | 1 |
| --- | --- |
| No | 0 |
| Unable to determine | 0 |

1. *Were the main outcome measures used accurate (valid and reliable)?*
   1. For studies where the outcome measures are clearly described, the question should be answered yes. For studies which refer to other work or that demonstrates the outcome measures are accurate, the ques- tion should be answered as yes.

| Yes | 1 |
| --- | --- |
| No | 0 |
| Unable to determine | 0 |

*Internal validity - confounding (selection bias)*

1. *Were the patients in different intervention groups (trials and cohort studies) or were the cases and controls (case-control studies) recruited from the same population?*
   1. For example, patients for all comparison groups should be selected from the same hospital. The question should be answered, unable to determine for cohort and case- control studies where there is no information concerning the source of patients included in the study.

| Yes | 1 |
| --- | --- |
| No | 0 |
| Unable to determine | 0 |

1. *Were study subjects in different intervention groups (trials and cohort studies) or were the cases and controls (case-control studies) recruited over the same period of time?*
   1. For a study which does not specify the time period over which patients were recruited, the question should be answered as unable to determine.

| Yes | 1 |
| --- | --- |
| No | 0 |
| Unable to determine | 0 |

1. *Were study subjects randomised to intervention groups?* Studies which state that subjects were randomised should be answered yes except where method of randomisation would not ensure random allocation. For example alternate allocation would score no because it is predictable.

| Yes | 1 |
| --- | --- |
| No | 0 |
| Unable to determine | 0 |

1. *Was the randomised intervention assignment concealed from both patients and health care staff until recruitment was complete and irrevocable?*

| Yes | 1 |
| --- | --- |
| No | 0 |
| Unable to determine | 0 |

1. *Was there adequate adjustment for confounding in the analyses from which the main findings were drawn?*
   1. *This question should be answered no for trials if: the main conclusions of the study were based on analyses of treatment rather than intention to treat; the distribution of known confounders in the different treatment groups was not described; or the distribution of known confounders differed between the treatment groups but was not taken into account in the analyses. In non-randomized studies if the effect of the main confounders was not investigated or con- founding was demonstrated but no adjustment was made in the final analyses the question should be answered as no.*

| Yes | 1 |
| --- | --- |
| No | 0 |
| Unable to determine | 0 |

1. *Were losses of patients to follow-up taken into account?*
   1. *If the numbers of patients lost to follow-up are not reported, the question should be answered as unable to determine. If the proportion lost to follow-up was too small to affect the main findings, the question should be answered yes.*

| Yes | 1 |
| --- | --- |
| No | 0 |
| Unable to determine | 0 |

*Power*

1. *Did the study perform calculations to determine sufficient power to detect a clinically important difference?*
   1. *Sample sizes have been calculated to detect a difference of x% and y%.*

| Yes | 1 |
| --- | --- |
| No | 0 |

***Total score: _____***
